# Transcriptomic Responses of the Heart and Brain to Anoxia in the Western Painted Turtle

**DOI:** 10.1371/journal.pone.0131669

**Published:** 2015-07-06

**Authors:** Sarah W. Keenan, Craig A. Hill, Cyriac Kandoth, Leslie T. Buck, Daniel E. Warren

**Affiliations:** 1 Department of Biology, Saint Louis University, St. Louis, Missouri, United States of America; 2 The Genome Institute, Washington University School of Medicine, St. Louis, Missouri, United States of America; 3 Department of Cell and Systems Biology, University of Toronto, Toronto, Ontario, Canada; The University of Texas Arlington, UNITED STATES

## Abstract

Painted turtles are the most anoxia-tolerant tetrapods known, capable of surviving without oxygen for more than four months at 3°C and 30 hours at 20°C. To investigate the transcriptomic basis of this ability, we used RNA-seq to quantify mRNA expression in the painted turtle ventricle and telencephalon after 24 hours of anoxia at 19°C. Reads were obtained from 22,174 different genes, 13,236 of which were compared statistically between treatments for each tissue. Total tissue RNA contents decreased by 16% in telencephalon and 53% in ventricle. The telencephalon and ventricle showed ≥ 2x expression (increased expression) in 19 and 23 genes, respectively, while only four genes in ventricle showed ≤ 0.5x changes (decreased expression). When treatment effects were compared between anoxic and normoxic conditions in the two tissue types, 31 genes were increased (≥ 2x change) and 2 were decreased (≤ 0.5x change). Most of the effected genes were immediate early genes and transcription factors that regulate cellular growth and development; changes that would seem to promote transcriptional, translational, and metabolic arrest. No genes related to ion channels, synaptic transmission, cardiac contractility or excitation-contraction coupling changed. The generalized expression pattern in telencephalon and across tissues, but not in ventricle, correlated with the predicted metabolic cost of transcription, with the shortest genes and those with the fewest exons showing the largest increases in expression.

## Introduction

Most vertebrate species can survive with little oxygen (hypoxia) or without oxygen entirely (anoxia) for only short periods of time, ranging from minutes in anoxia-sensitive mammals [1], to just a few hours in most ectothermic species at warmer temperatures (>20°C)[2, 3]. Pond turtles, however, especially those in the family Emydidae, can survive for at least a day of anoxia at 20–25°C [4], and one species, the western painted turtle, *Chrysemys picta bellii*, can survive for more than 30 hours at 22°C [5] and for 177 days at 3°C [6], making this species the most anoxia-tolerant tetrapod known. The physiological adaptations that account for this ability include: 1) profound metabolic depression, 2) large tissue glycogen stores, and 3) large extracellular buffering capacity afforded by the shell and skeleton, the latter of which prevents lethal decreases in body fluid pH that occur with extreme lactic acidosis [7–9].

In most vertebrates, the most anoxia-sensitive organs are the brain and heart, principally because of their high metabolic rates [10]. In anoxic turtles, both organs show changes in physiological function that result in energy savings. In the cerebrocortex, anoxia leads to suppression of spontaneous electrical activity (i.e., spike arrest) [11, 12], silencing of AMPA and NMDA receptors [13–16], and large GABA releases during anoxia that activate an inhibitory postsynaptic shunt and prevent presynaptic glutamate release [17, 18]. In the heart, a reduction in energy consumption is achieved mainly by depressed heart rate and contractility [19–22]. Despite our knowledge of the metabolic and physiological response of these organs to anoxic or hypoxic conditions, little is known about their transcription-level responses in these organs, especially the heart [23]. With the recent sequencing of the western painted turtle genome [23], an unprecedented degree of insight can now be gained into the transcriptomic response of turtle organs to anoxia. In the present study, we utilize this genomic data for three purposes: (1) to determine if there are any transcriptomic correlates with known physiological function during anoxia in the two best-functionally characterized organs in anoxic turtles, the brain and heart, (2) to identify new targets for future functional studies of cellular and organ function during anoxia, and (3) to test the hypothesis that the cost of transcription correlates with the pattern of gene expression observed during anoxia, when cells are energetically-starved.

## Materials and Methods

### Animals

Ten western painted turtle adults (N = 10; mean 238 g, range 198–274 g) of both sexes were purchased from a commercial vendor (Niles Biological Inc., Sacramento, CA), but were originally from Minnesota. Animals were held at 15–20°C on a 12:12 photoperiod for at least two weeks prior to the experiments. Animal health was monitored daily through visual inspection of swimming, basking, and feeding behavior. Individuals were fed at least three times a week with commercially-available turtle feed (Tetra ReptoMin) and were held in large, flow-through tanks (183 cm x 91 cm x 61 cm) with 45 cm of water containing St. Louis, MO municipal tap water. This study was carried out in strict accordance with the recommendations in the Guide for the Care and Use of Laboratory animals of the National Institutes of Health. All animal husbandry and experimental protocols were approved by the Saint Louis University Institutional Care and Use of Animals Committee (IACUC, protocol number 2198).

### Anoxia Experiments

Turtles were maintained at 19°C for 48 hours prior to experimentation. Five turtles were submerged in a 19°C aquarium (65 cm x 35 cm x 35 cm) filled with water that was previously bubbled with nitrogen to displace oxygen to undetectable levels when measured with a dissolved oxygen meter (YSI DO200). A plastic grate was placed at the surface of the tank to ensure continued submergence and to prevent access to the headspace above the aquarium. After 24 hours, their necks were quickly clamped underwater followed by rapid decapitation. The clamping was required to insure there was no change in the oxygenation state of the blood prior to tissue sampling. The plastron was immediately removed with a bone saw and blood samples were quickly obtained by cardiac puncture of the exposed ventricle and analyzed for blood gases, pH, and plasma lactate. The ventricle was removed from the turtle, blotted on sterile gauze to remove any blood, and freeze clamped with tongs precooled in liquid nitrogen. The brain case was opened with Rongeurs, and each half of the telencephalon removed, dissected of any visible white matter and meninges, and freeze-clamped. All tissues were stored at −80°C until used in RNA extractions. All dissections were carried out using aseptic technique.

### Blood-gas and Lactate Analyses

Blood gases tensions (partial pressure of O_2_ [Po_2_] and CO_2_ [Pco_2_], as mmHg) were measured using a Radiometer blood-gas analysis system (BMS MKII, PHM73 meter) and a modified pH measurement system as used previously [7]. All electrodes were thermostatted to 19°C. Blood was centrifuged at 9600 x g for 3 minutes and the plasma lactate analyzed using a commercially available kit (Trinity Biotech, Ireland). Blood parameters were statistically compared between tissues and treatments using unpaired t-tests or Mann-Whitney U-tests when data were non-parametric. Differences were significant when P < 0.05. All statistical computations were carried out using SigmaPlot 12.

### Tissue Analysis

Samples (50–100 mg) of frozen telencephalon and ventricle were ground to a fine powder in an autoclaved ceramic mortar and pestle that had been precooled with liquid nitrogen. The powder was transferred to dry-ice cooled, RNase-free, and sterile Eppendorf tubes. One milliliter of room-temperature TRIzol reagent (Life Technologies) per 50–100 mg of tissue powder was added, and the tubes were immediately vortexed. The remaining RNA isolation steps were performed according to the manufacturer's instructions and RNA concentration was measured with a spectrophotometer (Eppendorf BioPhotometer, Hamburg, Germany). The effects of anoxia on RNA contents within each tissue type were analyzed using Two-Way ANOVA and Student-Newman Keuls post-hoc tests. Statistical computations were carried out using SigmaPlot 12.

### mRNA Sequencing (mRNA-Seq)

The RNA pellets were resuspended in DEPC-treated water, treated with DNase I (Life Technologies) and RNA quality measured with a Bioanalyzer (Agilent 2100). The four samples with the highest RIN (RNA integrity number) values for each tissue and treatment (16 total samples) were used for RNA-seq. All RIN values for these samples were greater than 7.4 [24]. cDNA library construction and sequencing were carried out by the Washington University Genome Institute (St. Louis, MO), using previously described methods [25, 26] on an Illumina HiSeq 2000 platform with v3 chemistry. mRNA-Seq was carried out as described in Shaffer et al. [23]. In brief, paired-end 2x100 bp reads were generated from poly-A selected RNA-Seq libraries from 16 individually barcoded samples (8 telencephalon and 8 ventricle, 4 of each from anoxic and 4 from normoxic conditions). The sixteen samples were randomly distributed between two lanes in the flow cell. There was no significant difference between the number of paired-end reads between treatment groups or between tissue types (median total 2 x 100 bp reads per cDNA library = 50.4 million; Two Way ANOVA, F = 0.0818, P-value = 0.780, total df = 15). The painted turtle genome has a total of 241,320 exons averaging 298 bp in length (NCBI), resulting in an estimated exome size of 72 million base pairs (number of exons multiplied by their average length). Given a sampling depth of 50.4 million total reads (100 base pairs each), the dataset represents 70x coverage of the exome. Adapter sequences were screened with FLEXBAR v. 2.17.

To identify and quantify differentially expressed genes in response to anoxia in heart and brain, screened sequences were aligned to the *C*. *p*. *bellii* assembled reference sequence (version 3.0.1) using TopHat (version 1.4.0) [27]. TopHat splits reads to align them across known and novel splice junctions. To estimate transcript and gene abundances, Cufflinks 1.3.0 [28] was used, generating normalized FPKMs (Fragments Per Kilobase of exon model per Million mapped fragments) for each annotated gene and which was guided by a GTF (Gene Transfer Format) file of OPTIC annotations for known splice junction locations provided by the Ponting Laboratory (Oxford University, UK). The Cufflinks parameter-G was used to exclude novel isoforms, in order to exclude large outliers (regions with extraordinarily high read-depth) that introduce a loss of sensitivity to the Cufflinks normalization method. In rare case where the OPTIC gene model did not identify a human ortholog (just N = 8 transcripts), discontiguous megablast was used to query the model gene sequence in question against the human genomic plus transcript database (BLAST, NCBI) and identify the most likely human ortholog. The per-gene FPKMs were log_2_ transformed and compared across treatments and tissues using general linear model assuming a normal/Gaussian distribution [29] with FPR multiple testing correction using JMP Genomics 5.1 (ANOVA function within the software). Genes were excluded from the statistical analysis if the median FPKM equaled zero for three out of the four samples in each group under either treatment.

Mean FPKM and standard error of the mean (SEM) values were calculated for the four replicates from each tissue and treatment using FPKM values generated for each sample in Cufflinks. Sequence lengths and the number of exons for each differentially expressed gene were determined from the OPTIC-based annotation. Figures were generated using SigmaPlot 12. Change in FPKM reflects the difference between mean FPKM values under anoxic and normoxic conditions. The OPTIC-based annotation files used in this study are publicly available for download (http://figshare.com/articles/mm_cpicta3_gpipe_predictions_gft/1428637 and http://figshare.com/articles/c_picta_human_orthologs_with_id_prefix/1428635). Sequences for each of the 16 sequenced samples are available through NCBI (accession numbers SRS385157-71).

An analysis of gene ontology (GO) was conducted using freely available GO tools through the Lewis-Sigler Institute for Integrative Genomics (Princeton University, www.go.princeton.edu). Genes with increased expression levels were used as inputs for the analyses using the GOA human gene annotation as the reference database. Network analyses initially conducted with REViGO were exported to Cytoscape to generate figures of networks for statistically significant GO processes and functions. All results of GO analysis are presented as Supporting Information (S4–S9 Tables, S1–S3 Figs).

### Quantitative RT-PCR

As a way to validate the RNA-Seq results, the three genes with the most increased and decreased expression levels from each tissue were quantified using quantitative RT-PCR. DNase-treated RNA from each sample was reverse transcribed to cDNA (Omniscript, Qiagen) that was using qPCR (SYBR Green; Qiagen). Expression data are expressed as fold changes relative to two housekeeping genes (β2-actin and α1b-tubulin), which were unchanged in the RNA-Seq study, according to the ΔΔCT method [30, 31]. These housekeeping genes were selected because they were found to be unchanged in a previous study of gene expression during anoxia in turtle heart [32]. These were analyzed statistically using a one sample t-test (significance level P < 0.05). Statistical computations were carried out using SigmaPlot 12.

## Results

To investigate the effects of anoxia on the transcriptomic responses of the heart and brain of the anoxia-tolerant painted turtle, adult turtles were submerged in anoxic water for 24 hours at 19°C, and the ventricle and telencephalon were sampled and analyzed using RNA-seq. The P_O2_ of blood sampled by cardiac puncture at the end of the submergence was about 2 mmHg, indicating near-complete oxygen depletion. Blood Pco_2_ at the end of the submergence was increased to 65 mmHg and pH fell below 7, indicating respiratory and metabolic acidosis (Table 1). Plasma lactate increased significantly (Mann-Whitney RS Test, P-value = 0.029, T = 26, df = 6) in anoxic turtles, from a median of 2.62 ± 0.66 mM under normoxic conditions to 78.698 ± 7.59 mM in anoxic turtles.

**Table 1:** Blood gases and acid-base status of normoxic and anoxic painted turtles at 19°C.

	Po_2_ (mmHg) (N = 4)	Pco_2_ (mmHg) (N = 5)	pH (N = 5)
Normoxia	55.4 ± 16.5	26.8 ± 1.9	7.69 ± 0.02
24 hr Anoxia	2.3 ± 0.9	64.9 ± 6.3	6.96 ± 0.06

Values represent means ± SEM. N = number of individuals sampled per treatment group.

**Fig 1:**
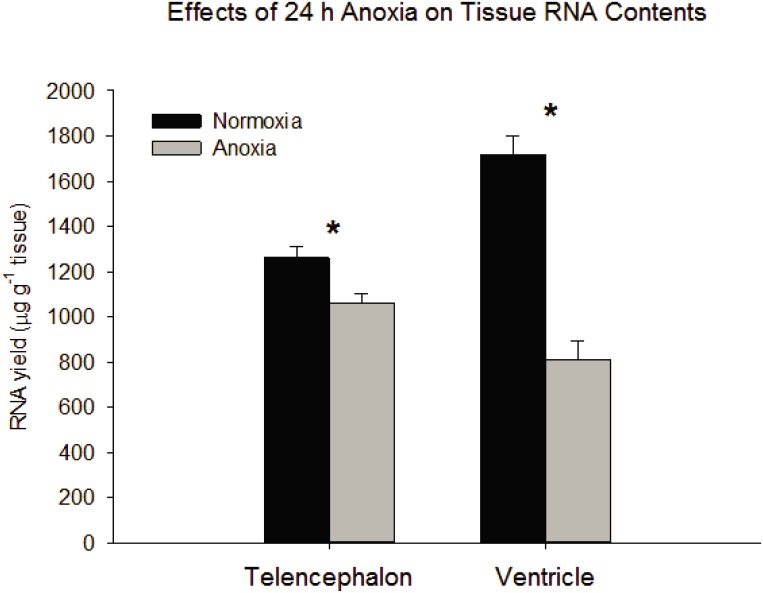
Effects of 24 hours of anoxia on tissue RNA content. After 24 hours of anoxia at 19°C, decreases in total tissue RNA occurred in both telencephalon and ventricle. * Indicates a significant difference between normoxia and anoxia within each tissue type. (Two-Way ANOVA, Tissue x Treatment df = 1, F = 28.303, P-value<0.001; SNK post-hoc, control v. anoxic telencephalon, q = 3.013, P-value = 0.049; SNK post-hoc, control v. anoxic ventricle, q = 13.653, P-value<0.001).

**Fig 2:**
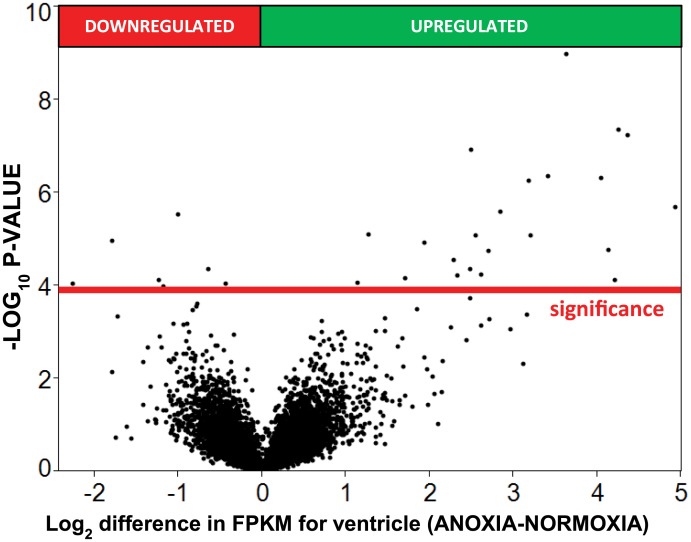
Volcano-plot showing transcriptomic changes in ventricle after 24 hours of anoxia at 19°C. The y-axis represents the −log10 of the P-value while the x-axis represents the log2 differential expression for each gene. Differential expression was determined using ANOVA assuming a log-normal distribution. The red reference line represents the significance threshold calculated from the FPR multiple testing correction.

**Fig 3:**
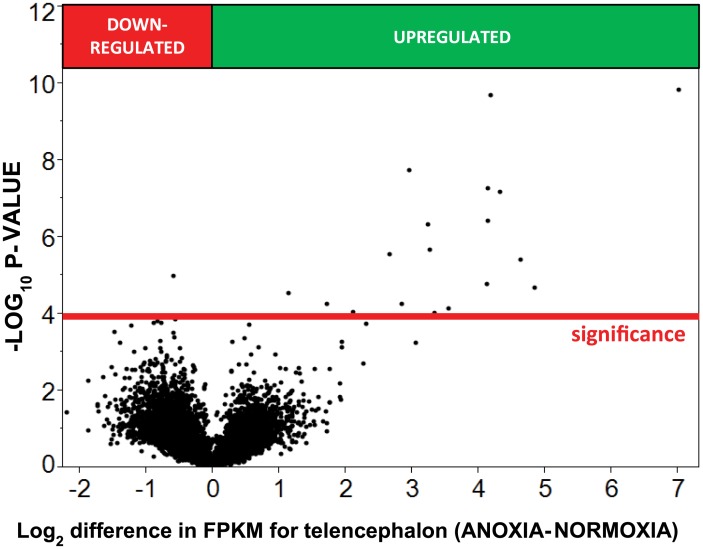
Volcano-plot showing transcriptomic changes in telencephalon after 24 hours of anoxia at 19°C. The y-axis represents the −log10 of the P-value while the x-axis represents the log2 differential expression for each gene. Differential expression was determined using ANOVA assuming a log-normal distribution. The red reference line represents the significance threshold calculated from the FPR multiple testing correction.

**Fig 4:**
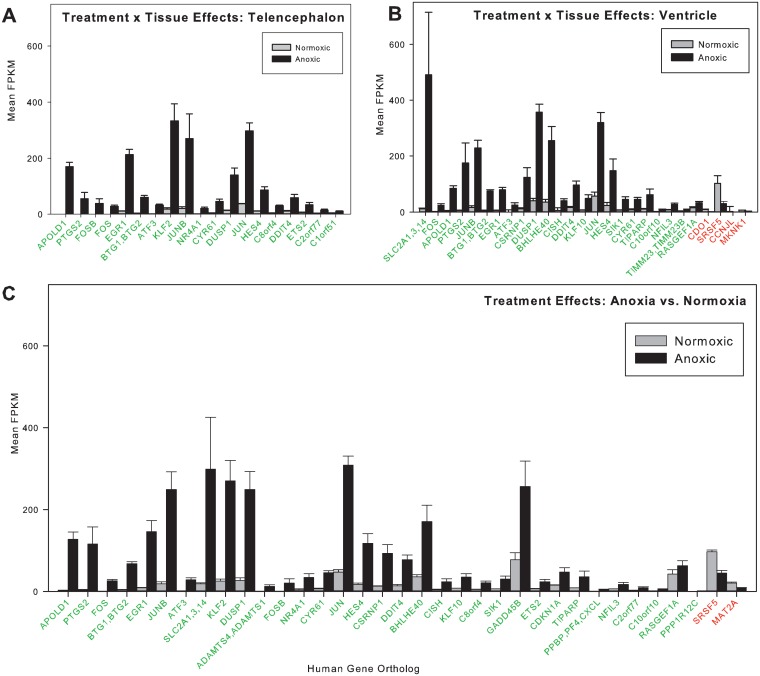
Mean ± SEM FPKM values measured for differentially expressed genes. (A) Values in normoxic and anoxic telencephalon, (B) normoxic and anoxic ventricle and (C) all normoxic and anoxic tissue combined. Genes with green font indicate increases in expression resulting from anoxia. Genes with red font indicate decreases in expression resulting from anoxia. In each plate, genes are arranged in order of decreasing fold expression. Only genes that changed by ≥ 2.0 fold or ≤ 0.5-fold were included. Several genes were abbreviated for simplicity: “PPBP,PF4,CXCL” includes Ppbp/Pf4/Cxcl1/Pf4v1/ Cxcl6/ Cxcl3/ Cxcl5/ Cxcl2/ Il8/ Cxcl10/ Cxc11; “CLK,PPIL3” includes Clk3/Ppil3/Clk1/Clk4). The mean FPKM values, P-values, sequence length, and exon density for each of these genes can be found in S1–S3 Tables.

Anoxic submergence decreased total tissue RNA content by 16% and 53% in telencephalon and ventricle, respectively (Fig 1). The reads from RNA-seq aligned to 22,174 human gene orthologs predicted from the OPTICS annotation file. After filtering genes such that only those where at least three of the four samples from each sampling group had FPKM values more than zero, 13,236 genes were compared statistically (Figs 2 and 3). In the telencephalon (Fig 4A) the expression of 19 genes increased (≥ 2x change), while no genes decreased (≤ 0.5x change). In the ventricle (Fig 4B), expression of 23 genes increased, while 4 decreased. The fold changes in tissue-specific gene expression have been published previously as supplemental material [23].

When only treatment effects (anoxia vs. normoxia) were examined across both tissues together, more changes in expression were observed than within each tissue on their own (Fig 4C). Under anoxic conditions, transcript levels increased in 31 genes (≥2x change) and decreased (≤ 0.5x change) in two genes.

**Fig 5:**
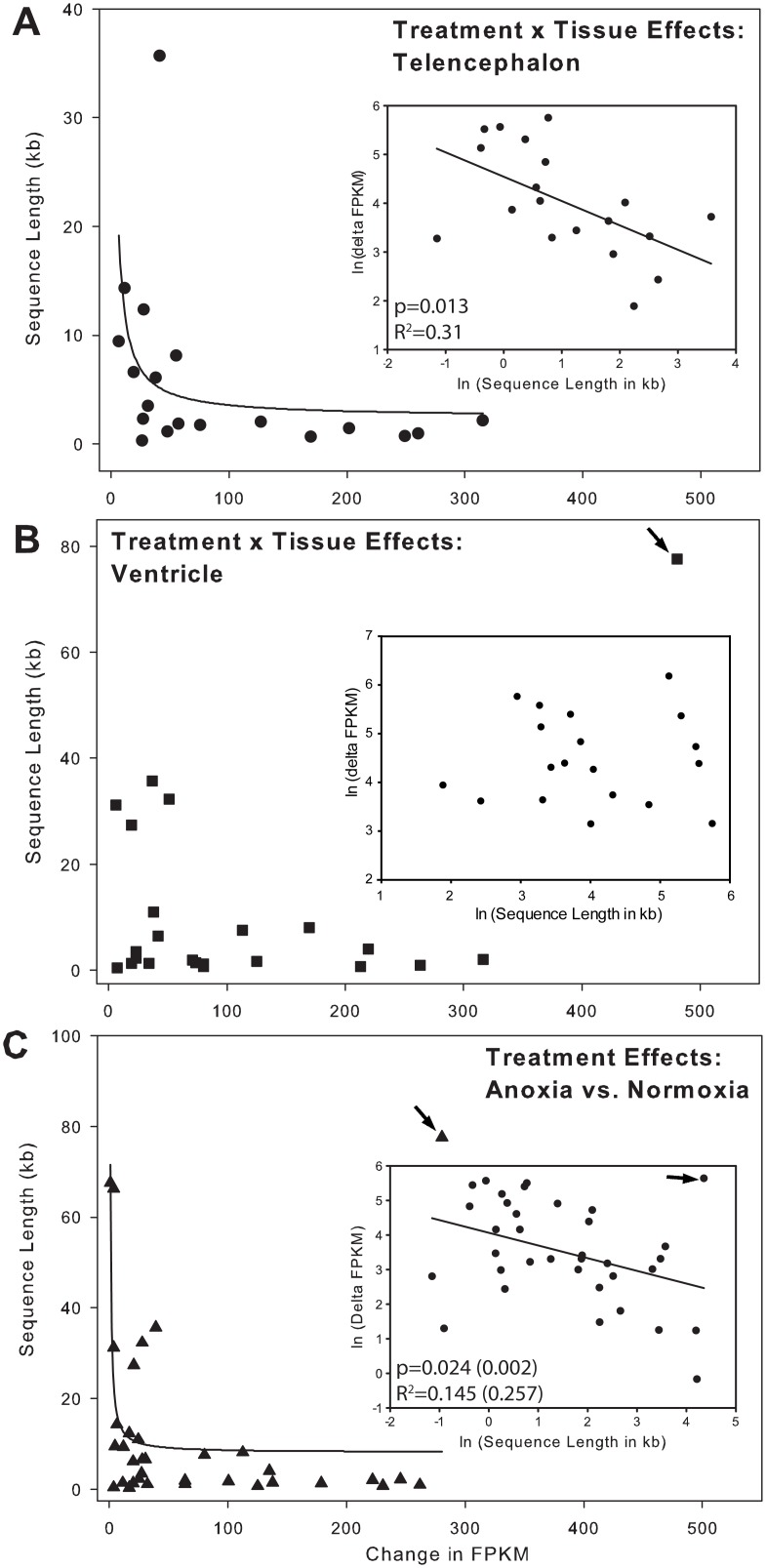
Sequence length as a predictor of increased gene abundance. (A) In telencephalon, longer genes show smaller changes in FPKM. The inset shows the linear regression analysis comparing the ln (delta FPKM) vs. ln (sequence length). (B) In ventricle, there is no significant relationship between delta FPKM and sequence length (regression not shown). The arrow indicates the expression value and sequence length of a GLUT isoform (Slc2a1,3,14). Excluding that point did not decrease the P-value appreciably. (C) When genes from all tissues were compared between treatments (treatment effects), sequence length is a significant predictor of delta FPKM. The values in parentheses indicate P-values and R2 values if the GLUT isoform (indicated by the arrowhead) is excluded from the linear regression analysis. Note: Although sequence length is the independent variable, it is shown on the y-axis to be consistent with reference [33]. The regression analyses (see insets in A and C) were conducted with ln (sequence length) as the independent variable. All significantly upregulated genes were included, regardless of fold-change.

**Fig 6:**
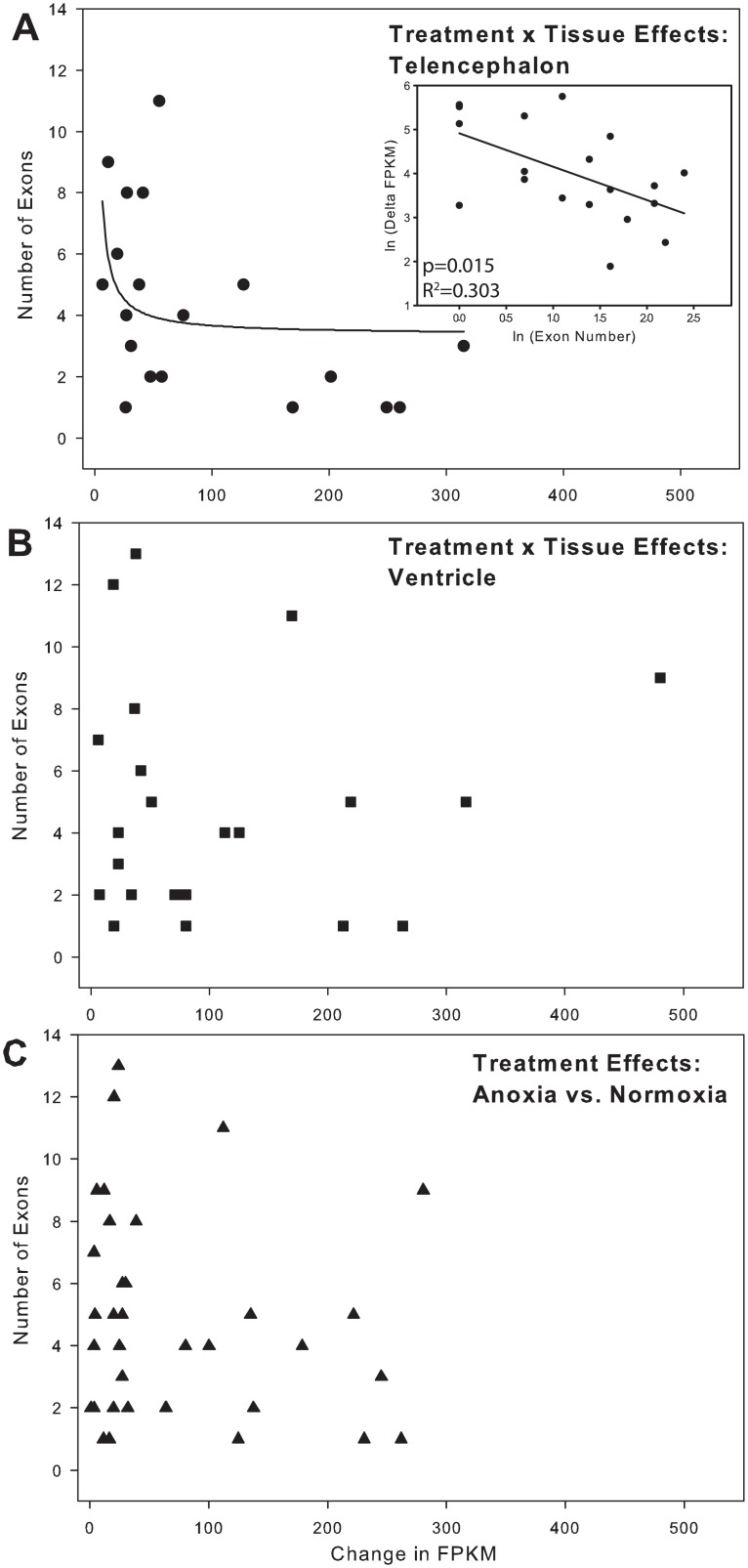
Exon number as a predictor of increased gene abundance. (A) In telencephalon, the number of exons in a gene was a significant predictor of the increase in the expression level of a gene. The inset shows that the ln (delta FPKM) decreases as the ln (Exon number) increases. (B) In ventricle alone and (C) when values from both tissue are pooled (treatment effect), ln (Exon number) is no longer a predictor of the ln (delta FPKM). Note: although sequence length is the independent variable, it is shown on the x-axis to be consistent with reference [33]. The regression analyses (see inset in A, not shown for B and C) were conducted with ln (Exon number) as the independent variable. All significantly upregulated genes were included, regardless of fold-change.

**Fig 7:**
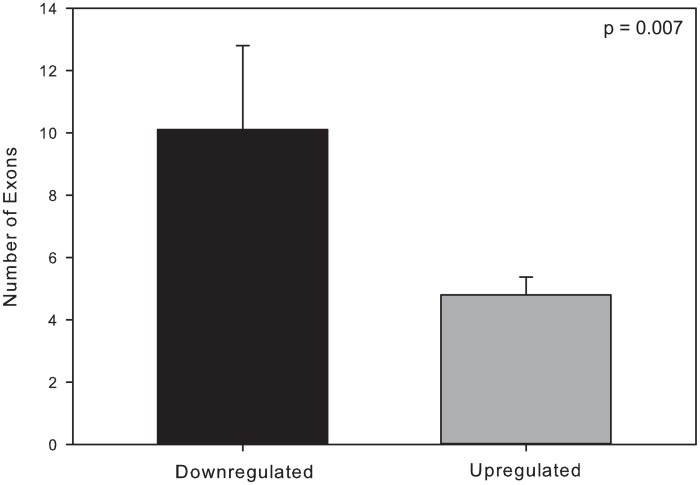
Exon number as a predictor of gene expression decrease. Downregulated genes had more exons than upregulated ones (t-test, P-value = 0.007, t = 2.848, df = 46). The genes used in this comparison were those found to be significantly different based on single factor (anoxia versus normoxia) general linear model across tissues (treatment effects only). Genes were included in this analysis regardless of their fold change. See the methods section for additional details surrounding this analysis.

Transcriptional costs are directly related to gene length and splicing. When the absolute change in FPKM values for each elevated transcript was compared to their sequence length (Fig 5), longer genes had the smallest change in FPKM values in telencephalon (Fig 5A) and between treatment values across tissues (Fig 5C). A similar relationship between exon density, a predictor of splicing requirement, and change in FPKM was also observed for telencephalon (Fig 6A), but not for ventricle (Fig 6B) or between the treatments of the two tissues (Fig 6C). Additionally, genes that significantly decreased expression during anoxia, across treatments, contained a significantly (t-test, P-value = 0.007, t = 2.848, df = 46) greater number of exons (mean = 10.1 ± 2.7) compared to increased genes (mean = 4.8 ± 0.56) (Fig 7).

In order to compare the RNA-seq results to conventional methods of quantifying gene expression, the expression levels of a subset of up-regulated and down-regulated genes from ventricle and telencephalon were also measured using the qPCR/ΔΔCT (S4 Fig). With the exception of a tendency for the qPCR to give higher fold-changes for the most highly expressed genes, the two methods were in general agreement and qualitatively similar.

## Discussion

Physiological stressors like anoxia result in an organismal response at all levels of biological organization, including mRNA abundance. Using tissues from adult painted turtles, the most anoxia-tolerant tetrapod known, our goals were to evaluate transcriptomic changes in response to anoxia to identify transcriptomic correlates of known physiological function, and to test the hypothesis that the costs of transcription might influence gene expression. Previous work has demonstrated significant physiological changes during anoxia, including spike arrest, silencing of AMPA and NMDA receptors, and large GABA releases in the brain [34], and reduced energy consumption in the heart. Based on the significant and diverse physiological responses to anoxia, genes directly correlating with known physiological traits were expected to be differentially expressed. However, the majority of the genes that changed were immediate early genes and related transcription factors, which are related genes known to upregulated in response to various forms of cellular stress, including hypoxia [35, 36].

While many of the changes in gene expression were observed in both the brain and heart, some were found exclusively in one tissue or the other. Hereafter, we discuss the changes that occurred in both tissues, followed by the changes that were unique to telencephalon and ventricle. We conclude with a consideration of gene expression patterns in the context of an energy limited system, independent of gene function.

### Changes Observed in Both Telencephalon and Ventricle

Of the 30 different genes whose levels increased during anoxia in this study, 12 increased in both telencephalon and ventricle. In tumors and other hypoxically stress tissues, many of the upregulated genes regulate vascular endothelial function [37]. It was, therefore, unsurprising to find that many of the genes that increased in the present are known to affect vascular endothelial function. This includes *Apold1*, an apolipoprotein that increased by more than 128-fold in telencephalon after anoxia, the most of any gene, and 30-fold in the ventricle. *Apold1*, also known as *Verge*, has been shown to be upregulated in mammalian vascular endothelial cells in response to hypoxia and may be important in regulating the blood-brain barrier [38]. Other increased genes known to change in vascular endothelium in mammals with hypoxia, and which were upregulated in the present study, include *Erg1* [39] and *Ptgs2* [40–42].

Increased expression of *Cyr61*, 5.0-fold and 9.7-fold in the ventricle and telencephalon after anoxia, respectively, is also associated with endothelial cells. In mice and humans, expression of *Cyr61* is linked to increased angiogenic activities and wound healing [43, 44] and is hypoxia-inducible in mammals [45]. Enhanced vascularization and over-expression of *Cyr61* are also implicated in tumor growth [35], where conditions are predominantly hypoxic [46].

In addition, *Fos* expression increased in both ventricle (20.7-fold) and telencephalon (20.1-fold). The mRNA and protein expression of *c-Fos* has been previously shown to increase in anoxic red-eared slider turtle brain [47, 48], but expression of protein decreases in slider turtle heart [47], a difference that may either be related to species, experimental temperature, and/or translational effects. *Fos* is an immediate early gene associated with cell proliferation and differentiation that changes expression pattern under a range of environmental stimuli, including auditory stimuli in the zebra finch brain [49], and stresses (e.g., ischemia and hypoxia) [47]. *Jun* and *JunB*, additional immediate early genes often associated with *Fos*, also increased in both tissues, with fold-changes of 5.6 and 17.6 in the ventricle and 7.8 and 11.7 in the telencephalon following exposure to anoxic conditions. Expression of *Jun* family genes is associated with cell differentiation, apoptosis, and proliferation in normal, healthy tissues as well as in cancerous cells [50]. Both *Jun* and *Fos*, in addition to several other genes not differentially expressed in these experiments (i.e. *Bcl-X*, *Bcl2*, *Hsp70*), have previously been described as hypoxia inducible genes [48]. Their increase in the previous study of sliders, as well as this study in painted turtles, suggests that these gene transcripts provide a critical and adaptive role in the ability of turtles to survive anoxia.

*Activating transcription factor 3* (*Atf3*) expression also increased in both tissues (10.6 fold change in ventricle and 17.6 in telencephalon after anoxia). *Atf3* upregulation occurs in response to a variety of physiological stressors, and has the potential to heterodimerize with *Jun* family proteins, activating transcription of multiple genes [51]. In mammals, physiological stress, including heart ischemia, results in increased *Atf3* expression [51]. Therefore the observation of increased *Atf3*, as well as *Jun* and *Fos* family gene expression fits within our current understanding of genes associated with hypoxic stress in vertebrates, and suggests that these genes play a critical, and probably protective role, in the immediate and early responses of an organism to oxygen-limitation stress.

Other genes that increased in both ventricle and telencephalon include anti-proliferation genes (*Btg1/2*, *Dusp1*), *DNA damage inducible transcript 4* (*Ddit4*), and *Hes4*. *Ddit4* was previously observed to increase in tissues exposed to hypoxic conditions in humans [52]. Increased *Btg1* expression was observed in the goby (*Gillicthyes mirabilis*), a hypoxia-tolerant fish, where it was suggested to play a role in preventing cellular growth and reducing energetic demands [53]. In the present study, *Btg1/2* exhibited a 17.1 fold-change in expression in anoxic conditions, the fourth largest fold-change of the 48 differentially expressed genes. The role of *Btg1/2* in preventing cell growth suggests that the observed increase in expression in anoxic painted turtles in this study is an adaptive feature and may be present across a great diversity of vertebrates.

The heart, and particularly the brain, are heterogeneous [54] assemblies of cell types with variable densities, distributions, and functions. This makes an organ-scale transcriptomic response essentially an average of all individual cellular changes. In fact, our basic understanding of the transcriptomics response(s) of each cell type to anoxia is far from understood for any vertebrate system. Undoubtedly, there are cell-specific responses; however, this has yet to be explored in depth, and provides a potentially fruitful avenue for future research through the use of *in situ* hybridization.

### Changes Unique to Telencephalon

Seven of the 19 upregulated genes found to be differentially expressed were unique to the telencephalon. These included *FosB*, *Nr4a1*, *Ets2*, one gene similar to *Klf2* in *Xenopus*, and three open reading frames (orfs, including *C8orf4*), two with no known function. *FosB* dimerizes with *Jun* to form transcription factor AP-1, which itself, activates a variety of immediate early genes believed to promote survival in the nervous system [55]. *Nr4a1* is a nerve growth factor involved in inflammation and apoptosis [56]. *Ets2* is a transcription factor that regulates telomerase activity and may function to help protect chromosomes from damage [57, 58]. *C8orf4*, also known as *Tc1*, induces heat-shock proteins [59], which were upregulated during anoxia in a previous study of painted turtle brain [60]. *Klf2* is important in maintaining vascular endothelial function in liver [61].

### Changes Unique to Ventricle

In the ventricle, 11 of the 26 upregulated genes were unique to this tissue, including a glucose transporter gene that closely resembles human GLUTs 1,3 and 14. The GLUT isoforms increased by nearly 31-fold after anoxia and showed the largest increase in expression level in the ventricle. A fold-change this large suggests that the ventricle is preparing to increase the transport of either glucose or dehydroxyascorbic acid (DHA)[62], both of which would be adaptive during anoxia, as glucose transport must be maintained to meet glycolytic demands. An increase in DHA transport would be useful in scavenging reactive oxygen species during the reperfusion that takes place during recovery [63].

Other genes with increased expression in anoxic conditions unique to ventricle included *Csrnp1*, or *Axud1*, whose function is unknown but may be involved in tumor suppression [64]. *Bhlhe40*, *Klf10*, *Nfil3* (also known as *E4bp4*) are all transcriptional repressors that were increased in ventricle [65, 66]. *Cish*, which is an inhibitor of the JAK/STAT signaling pathway, is induced by *Epo*. *Sik1* increases Na/K ATPase activity [67], but has no known physiological correlate in turtles. *Tiparp* is involved in protein ribosylation [68] and could be cardioprotective. *C10orf10*, also known as *Depp*, is upregulated by hypoxia in murine kidney and brain [69]. *Timm23*, a mitochondrial protein translocase, and RasGEF, a nucleoside exchange protein, were also upregulated in turtle ventricle.

The ventricle was the only tissue to show decreases in gene expression during anoxic conditions, as four genes were identified as statistically different, including *Cdo1*, a regulator of intracellular cysteine levels. This is particularly interesting because *Cdo1* knockout mice show increased hydrogen sulfide production [70], which is a known metabolic depressant [71]. *Srsf5*, an mRNA splicing factor, was also decreased, along with *Mknk1*, which normally interacts with *Mapk1* to stimulate translation. Additionally, cyclin J (*CcnJ*), which is a cell cycle gene highly expressed in some tumors [72], decreased during anoxia. Taken together, the decreases in ventricular gene expression would seem to promote metabolic depression and translational arrest.

### mRNA Expression Patterns in an Energy-Limited System

The anoxic turtle affords the opportunity to examine how transcript abundance patterns might reflect the energetic cost of transcribing genes in tissues known to be sensitive to hypoxia in vertebrates, including humans. Previous work has highlighted the connection between gene expression and intron length, with the most highly expressed genes tending to have the shortest introns [33]. The costs of transcription are directly dependent on the number of nucleotides within the genes, as well as the number of splice events to produce the final translatable mRNA. Under anoxic conditions, where energy is limited, it would be expected that: 1) any genes that are upregulated are likely to be inexpensive to produce (i.e. short sequences with few exons), and 2) any upregulated genes that are either long or have many exons must have an importance directly related to some product of their expression cost and their copy number. In the present study, it was found that in telencephalon, specifically, and across tissues, generally, that the shortest genes show the largest increase in expression level during anoxia (Fig 5A, 5C), with the exception of a single outlier, a gene coding for the overexpressed GLUT isoform. It is tempting to infer that the relative importance of the expression of the GLUT isoform for turtle survival during anoxia is high because of the metabolic cost of transcribing such a long gene. At this time, however, we cannot rule out the possibility that this is a simple outlier or the product of the new annotation. When the analysis is extended to the number of exons in the genes, the number of exons in downregulated genes is significantly higher than the number in upregulated genes (Fig 7; t-test, P-value = 0.007, t = 2.848, df = 46). In this analysis, all genes found to be significantly different were included, regardless of fold-change. Genes whose expression decreased had more exons than those that were increased. Based on these analyses, it may be that, in some tissues, genomic architecture (i.e. sequence length and intron/exon density) within a set of highly conserved “adaptive” genes could determine their own expression level due to the energetic cost of their transcription.

## Conclusions

The present study demonstrates that adult painted turtles show loss of tissue RNA during anoxia, which points to ribosomal degradation as the most likely mechanism of translational arrest in telencephalon and, especially, ventricle. Despite a decrease in tissue RNA, there were large increases in gene expression levels in both tissues. However, none of these genes were involved in excitatory aspects of neuronal or muscle function. Instead, many have been previously implicated as immediate early genes and transcriptional, translational, or metabolic repressors. The upregulation of some genes associated with enhanced vascularization in epithelial tissue (e.g., *Cyr61*, *Apold1*) observed in both telencephalon and ventricle suggests that increasing gene transcripts directly related to vascular endothelial function may also be an adaptive feature for surviving anoxia. However, the similarity in overall transcriptomic landscape of samples derived from the same tissue, regardless of treatment conditions (Fig 4) suggests that the response of the painted turtle that affords anoxia tolerance affects a narrow and adaptive set of genes. The function of these genes in painted turtles and the timing of transcript abundance changes during anoxia remain largely unknown.

Our finding that relatively few genes were decreased in the present study warrants further examination and might have resulted either from low sample size (N = 4 per group) or, more likely inadequate sequencing depth. Our conservative approach of excluding genes in which three of four samples had zero FPKM might have resulted in an underestimation of decreased gene expression. Large reductions in gene expression to undetectable levels would have yielded many genes with zero counts and, therefore, increase the likelihood they were excluded from the analysis. This reflects an inherent bias in RNAseq studies, which is to count only the most highly expressed transcripts.

Future RNA-seq studies of anoxia in turtles should be strand-specific to reveal non-coding RNAs from the anti-sense strand, and should sequence to a higher coverage depth to obtain a larger sampling of down-regulated genes. Additionally, as the organs examined in this study are composed of multiple cell types, the observed changes in transcript abundance are likely to provide an average organ-scale response. However, the changes in transcript abundance at the cellular level require further investigation, including whether expression changes are uniform between cells and within a single organ, because the potential for heterogeneous organ-scale response is high. This is supported by our observation that many of the upregulated genes in both organs are known to change in vascular endothelium, rather than the functional cell type normally associated with the function of that organ (eg, neuron or myocyte). To establish functional relevance, gene expression validation at the protein level is needed, along with ChIP-seq studies of the overexpressed transcription factors. Finally, additional analyses of the energetic costs of gene expression should be taken into consideration, as they may inform us of whether a change in gene expression is important in an energy-starved state.

## Supporting Information

S1 Fig (A) Functional and (B) process-based gene ontology network analyses of genes with increased expression under anoxic conditions from telencephalon. Smaller, darker nodes correlate to the lowest P-values measured for the set of GO terms. Branches between nodes reflect predicted network associations, with the thickness of branches reflecting strength of association.
(PDF)

S2 Fig (A) Functional and (B) process-based gene ontology network analyses of genes with increased expression under anoxic conditions from ventricle. Smaller, darker nodes correlate to the lowest P-values measured for the set of GO terms. Branches between nodes reflect predicted network associations, with the thickness of branches reflecting strength of association.
(PDF)

S3 Fig (A) Functional and (B) process-based gene ontology network analyses of genes with increased expression under anoxic conditions across tissues. Smaller, darker nodes correlate to the lowest P-values measured for the set of GO terms. Branches between nodes reflect predicted network associations, with the thickness of branches reflecting strength of association.
(PDF)

S4 Fig FPKM fold changes between treatments versus fold changes obtained from conventional qPCR methods. (A) Values obtained from the ventricle for two sets of housekeeping genes, α-tubulin (grey) and β-actin (black). (B) Additional comparisons between fold changes observed with RNA-Seq and qPCR using the same two housekeeping genes in telencephalon. The black line represents the line of identity in both plates. In both tissues, there was a tendency for qPCR to show higher fold changes than RNA-seq.
(PDF)

S1 Table Mean FPKM values of genes that changed significantly in painted turtle telencephalon after 24 hours of anoxia at 19°C. (PDF)

S2 Table Mean FPKM values of genes that changed significantly in painted turtle ventricle after 24 hours of anoxia at 19°C. (PDF)

S3 Table Mean FPKM values of genes that changed significantly across tissues in painted turtle across tissues after 24 hours of anoxia at 19°C. (PDF)

S4 Table Gene Ontology (GO) process-based outputs from genes that were significantly increased in telencephalon of anoxic painted turtles. (PDF)

S5 Table Gene Ontology (GO) process-based outputs from genes that were significantly increased in ventricle of anoxic painted turtles. (PDF)

S6 Table Gene Ontology (GO) process-based outputs from genes that were significantly increased across tissues (treatment only effects) in anoxic painted turtles. (PDF)

S7 Table Gene Ontology (GO) function-based outputs from genes that were significantly increased in telencephalon of anoxic painted turtles. (PDF)

S8 Table Gene Ontology (GO) function-based outputs from genes that were significantly increased in ventricle of anoxic painted turtles. (PDF)

S9 Table Gene Ontology (GO) function-based outputs from genes that were significantly increased across tissues (treatment only effects) in anoxic painted turtles. (PDF)
