# Supplementary figures and images for: Transcriptomic Responses of the Heart and Brain to Anoxia in the Western Painted Turtle
Source: PLoS One. 2015 Jul 6;10(7):e0131669. doi: 10.1371/journal.pone.0131669 (PMC4493013; doi:10.1371/journal.pone.0131669)

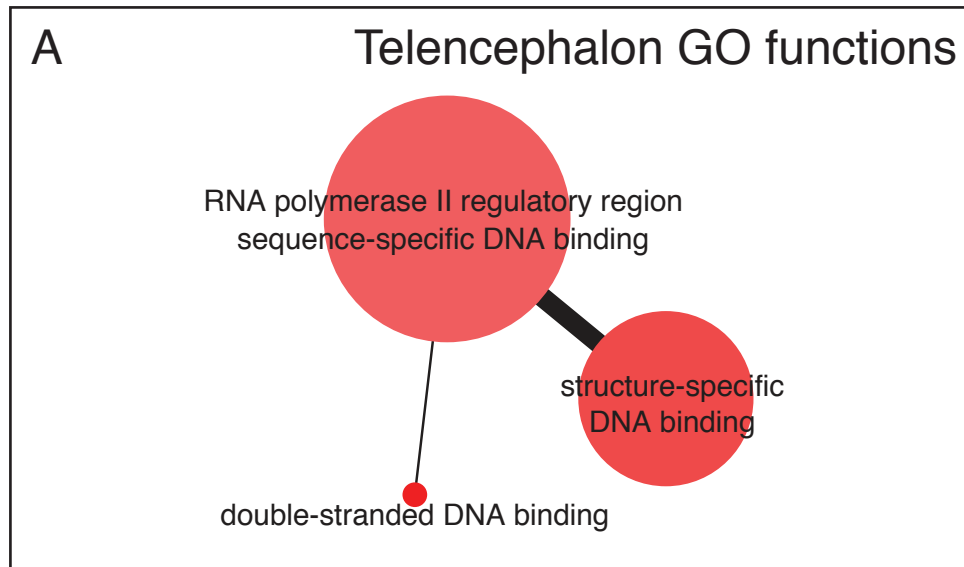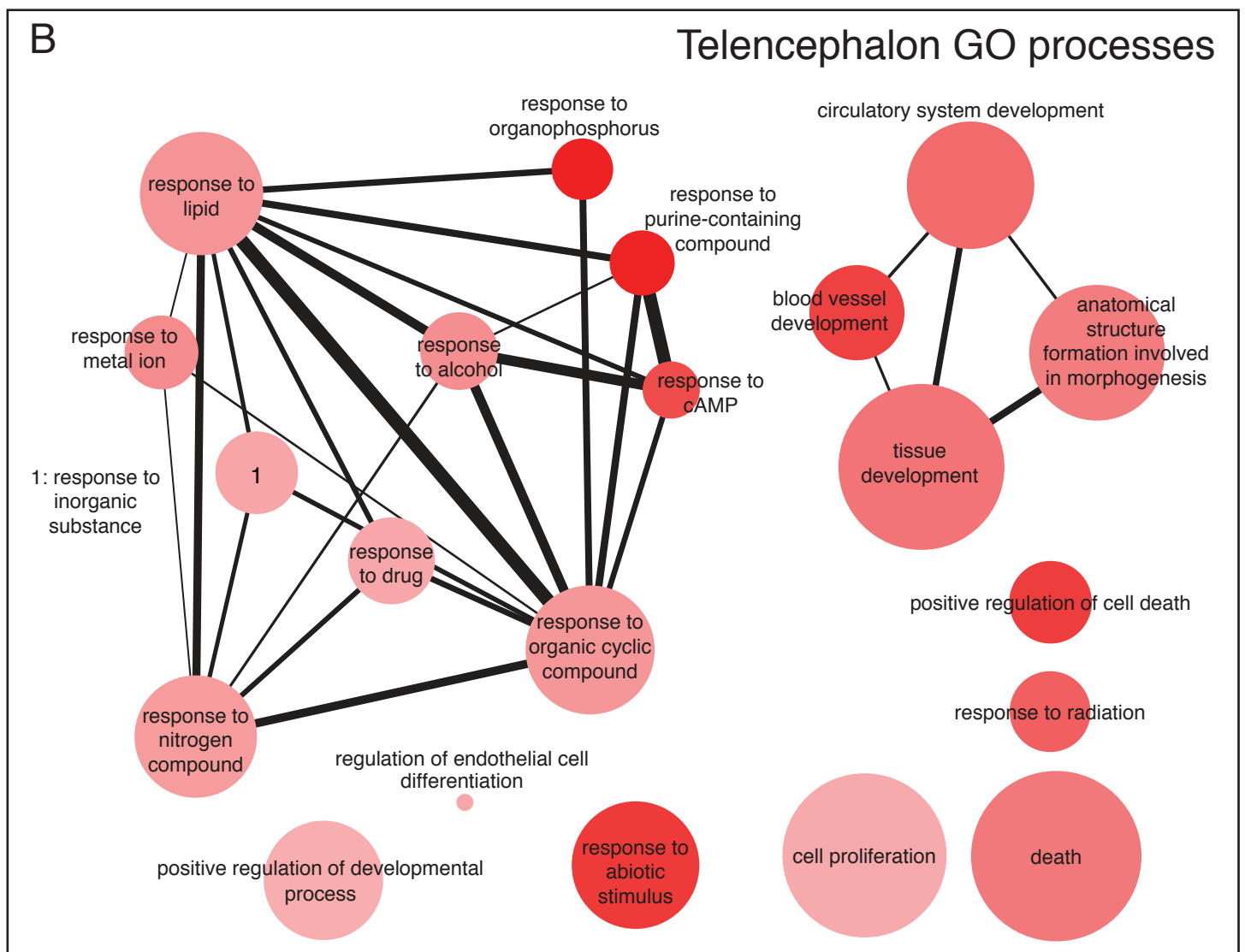

Supplement: S1 Fig — Smaller, darker nodes correlate to the lowest P-values measured for the set of GO terms. Branches between nodes reflect predicted network associations, with the thickness of branches reflecting strength of association. (PDF) [file pone.0131669.s001.pdf]

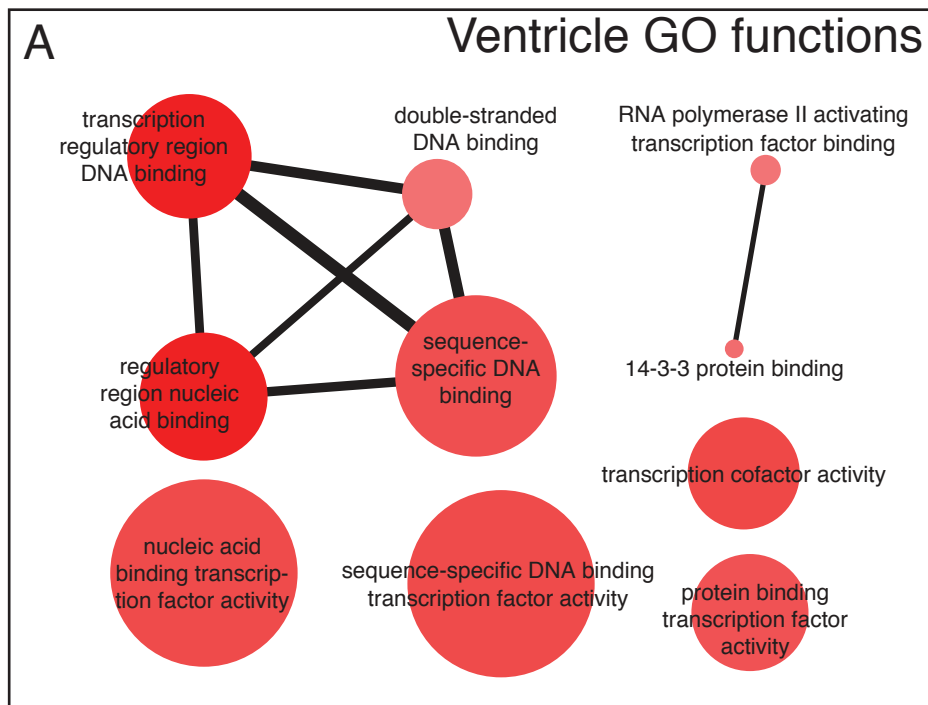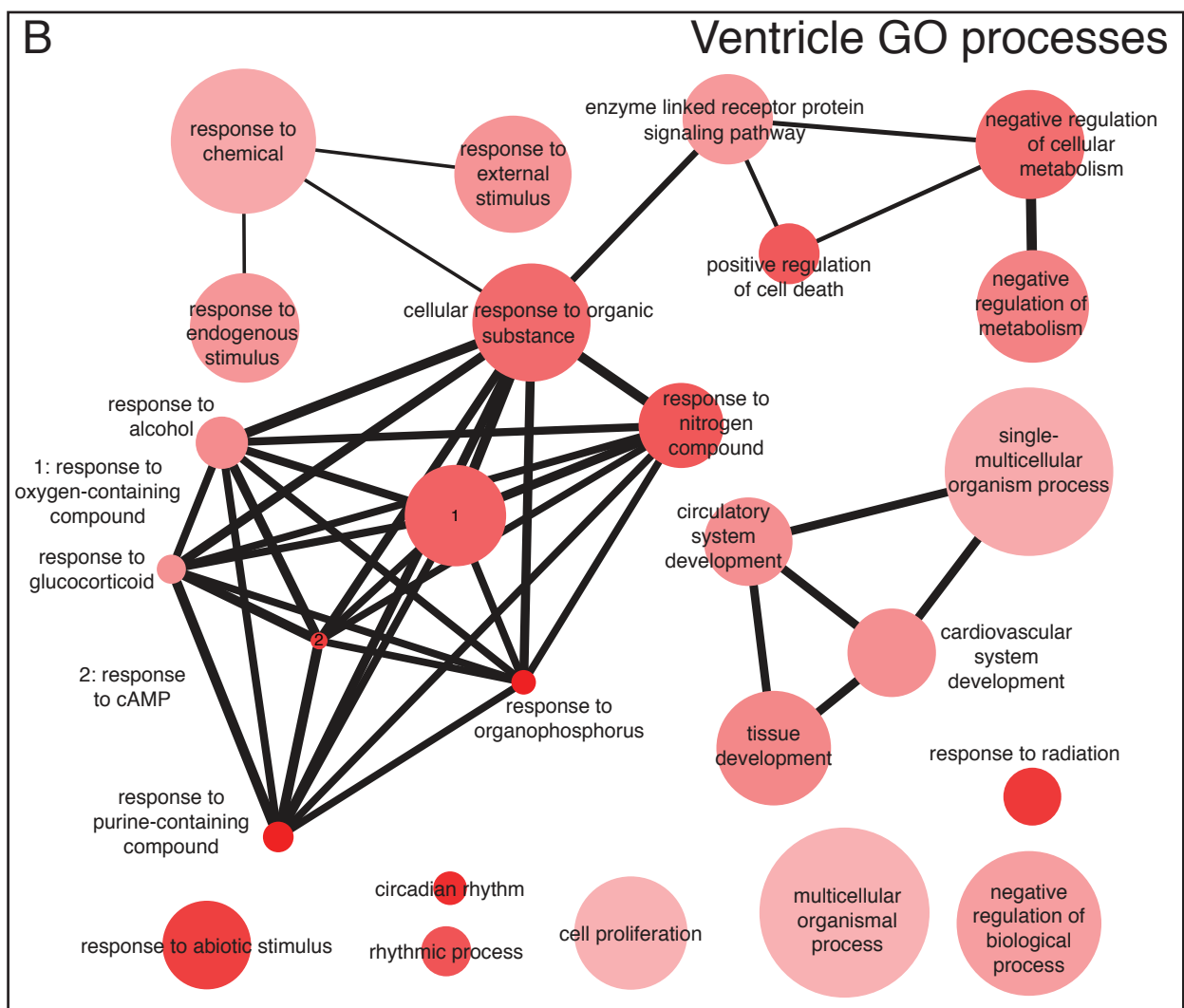

Supplement: S2 Fig — Smaller, darker nodes correlate to the lowest P-values measured for the set of GO terms. Branches between nodes reflect predicted network associations, with the thickness of branches reflecting strength of association. (PDF) [file pone.0131669.s002.pdf]

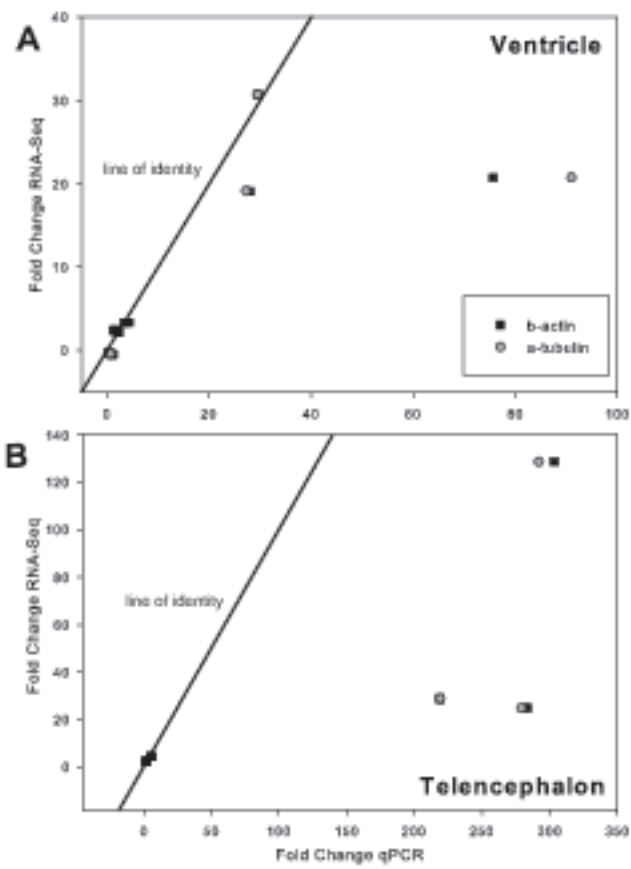

Supplement: S4 Fig — (A) Values obtained from the ventricle for two sets of housekeeping genes, α-tubulin (grey) and β-actin (black). (B) Additional comparisons between fold changes observed with RNA-Seq and qPCR using the same two housekeeping genes in telencephalon. The black line represents the line of identity in both plates. In both tissues, there was a tendency for qPCR to show higher fold changes than RNA-seq. (PDF) [file pone.0131669.s004.pdf]
