# Supplementary material for: Transcriptomic Responses of the Heart and Brain to Anoxia in the Western Painted Turtle
Source: PLoS One. 2015 Jul 6;10(7):e0131669. doi: 10.1371/journal.pone.0131669 (PMC4493013; doi:10.1371/journal.pone.0131669)
Supplement: S1 Table — (PDF) [file pone.0131669.s005.pdf]

S1 Table. Mean FPKM values of genes that changed significantly in painted turtle telencephalon after 24 hours of anoxia at 19°C.

Human Gene Ortholog	Sequence Length (kb)	# Exons	Mean FPKM ± SEM		$-LOG_{10}$(p-value)
			Normoxia	Anoxia	
APOLD1	0.677	1	0.3162 ± .0869	169.4843 ± 15.3225	9.837718
PTGS2	8.11	11	0.4858 ± .1754	55.6362 ± 55.6362	4.687669
FOSB	6.092	5	0.1692 ± .0839	38.0412 ± 38.0412	5.421253
FOS	2.301	4	0.3584 ± .2090	27.3408 ± 27.3408	7.185112
EGR1	1.45	2	10.8166 ± 1.2077	212.4740 ± 18.7898	9.69016
BTG1/2	1.878	2	2.6001 ± .6744	59.7873 ± 7.2043	6.423472
ATF3	3.504	3	0.8595 ± .1727	32.0300 ± 4.1616	7.266755
KLF2	2.154	3	18.3261 ± 3.8814	333.3068 ± 60.279	4.780772
JUNB	0.719	1	20.8766 ± 5.5343	269.846 ± 87.6450	4.139213
NR4A1	6.608	6	0.8967 ± .2356	20.0767 ± 4.8369	4.024999
CYR61	35.668	8	3.8738 ± .9033	45.0959 ± 8.6998	5.660014
DUSP1	2.056	5	13.2768 ± 1.6567	140.1647 ± 25.1123	6.329801
JUN	0.941	1	36.7934 ± 1.7867	297.1345 ± 29.0648	7.747229
HES4	1.75	4	10.9939 ± 1.3533	86.4969 ± 12.2511	4.253441
C8orf4	0.317	1	4.0971 ± .6818	30.5262 ± 7.7812	5.552381
DDIT4	1.155	2	10.9071 ± 2.2153	58.5520 ± 12.2882	3.747429
ETS2	12.372	8	6.6482 ± .9053	34.2827 ± 7.7812	4.051485
C2orf77	14.315	9	3.644 ± .4603	15.0211 ± 3.3850	4.270075
C1orf51	9.43	5	4.4468 ± .5173	11.0608 ± 1.2657	4.531651

Note: Only genes that changed by 2x or more were included. No genes changed by less than 0.5x. General linear model after log_2 transformation and normal/Gaussian distribution with FPR multiple testing correction procedure (ANOVA function in JMP Genomics 5.1).
