# Supplementary material for: Transcriptomic Responses of the Heart and Brain to Anoxia in the Western Painted Turtle
Source: PLoS One. 2015 Jul 6;10(7):e0131669. doi: 10.1371/journal.pone.0131669 (PMC4493013; doi:10.1371/journal.pone.0131669)
Supplement: S2 Table — (PDF) [file pone.0131669.s006.pdf]

S2 Table. Mean FPKM values of genes that changed significantly in painted turtle ventricle after 24 hours of anoxia at 19°C.

Human Gene Ortholog	Sequence Length (kb)	# Exons	Mean ± FPKM		$-LOG_{10}$(p-value)
			Normoxia	Anoxia	
SLC2A1,3,14	77.569	9	10.8976 ± 2.8492	491.3548 ± 222.4742	5.691103
FOS	2.301	4	0.0818 ± 1.5643	23.2370 ± 5.4786	7.229849
APOLD1	0.677	1	4.0291 ± 1.3826	84.5368 ± 9.1337	7.353036
PTGS2	8.11	11	5.8045 ± 1.0732	175.1762 ± 72.2778	4.115865
JUNB	0.719	1	15.8886 ± 5.8568	228.6000 ± 27.6852	4.762765
BTG1,2	1.878	2	4.41 ± 1.7527	75.2422 ± 4.3103	6.321031
EGR1	1.45	2	5.4819 ± 1.4316	79.3151 ± 8.8618	8.985092
ATF3	3.504	3	1.1155 ± 6.4759	24.4059 ± 8.0365	6.35429
CSRNP1	7.58	4	10.8884 ± 4.6775	123.7054 ± 35.4689	5.07269
DUSP1	2.056	5	40.2962 ± 7.9196	357.0168 ± 28.4303	6.255484
BHLHE40	4.023	5	35.8574 ± 8.8170	255.2610 ± 49.9381	5.59404
CISH	1.276	2	5.4282 ± 10.1909	39.7299 ± 7.0519	4.7544
DDIT4	1.155	2	16.3178 ± 4.0154	96.2664 ± 14.6133	4.227489
KLF10	6.512	6	6.9491 ± 1.4146	48.8401 ± 13.3949	5.085144
JUN	0.941	1	56.9791 ± 14.3643	320.2723 ± 36.0893	6.917876
HES4	1.75	4	22.9757 ± 11.4113	147.9281 ± 41.9951	3.730261
SIK1	11.013	13	6.6753 ± 1.1011	44.5425 ± 10.0939	4.343698
CYR61	35.668	8	8.4451 ± 2.4833	45.4374 ± 7.05110	4.216038
TIPARP	32.27	5	10.1792 ± 2.1944	61.4706 ± 20.0330	4.547673
C10orf10	0.404	2	1.4741 ± .3401	8.6225 ± 1.6285	4.919237
NFIL3	1.382	1	7.1885 ± 1.8733	26.7084 ± 5.3694	4.148264
TIMM23,23B	31.216	7	3.2877 ± 1.1184	9.4012 ± 0.9709	5.097863
RASGEF1A	27.391	12	14.5375 ± 3.6274	33.5824 ± 4.4896	4.056645
CDO1	12.435	5	8.9316 ± 1.3605	1.4726 ± 1.0026	4.047656
SRSF5	3.797	7	102.0765 ± 27.1269	30.7898 ± 6.8046	4.962089
CCNJL	43.753	9	1.8739 ± 17.5918	0.2716 ± 0.1073	3.981737
MKNK1	31.455	15	6.3689 ± 0.5484	2.6705 ± 0.1359	5.533251

Note: Only genes that changed by 2x or more and 0.5x or less were included. Italicized genes decreased expression levels.

General linear model after log_2 transformation and normal/Gaussian distribution with FPR multiple testing correction procedure (ANOVA function in JMP Genomics 5.1).
