# Supplementary material for: Transcriptomic Responses of the Heart and Brain to Anoxia in the Western Painted Turtle
Source: PLoS One. 2015 Jul 6;10(7):e0131669. doi: 10.1371/journal.pone.0131669 (PMC4493013; doi:10.1371/journal.pone.0131669)
Supplement: S3 Table — (PDF) [file pone.0131669.s007.pdf]

S3 Table. Mean FPKM values of genes that changed significantly across tissues in painted turtle across tissues after 24 hours of anoxia at 19°C.

Human Gene Ortholog	Sequence Length (kb)	# Exons	Mean ± FPKM		-LOG$_{10}$(p-value)
			Normoxia	Anoxia	
APOLD1	0.677	1	2.1727±0.9506	127.0105 ± 18.0528	10.487
PTGS2	8.110	11	3.1451±1.0347	115.4062 ± 41.6088	5.915048
FOS	2.301	4	0.2201±0.1105	25.2889 ± 3.6354	8.914674
BTG1,2	1.878	2	3.5051±1.0398	67.5148 ± 4.8614	8.041433
EGR1	1.450	2	8.1492±1.2024	145.8946 ± 26.9396	11.11215
JUNB	0.719	1	18.3826±4.5407	249.2230 ± 43.2558	5.971382
ATF3	3.504	3	0.9875±0.1295	28.2180 ± 4.4302	8.519981
SLC2A1,3,14	77.569	9	17.9621±2.8360	298.4417 ± 126.6830	5.607408
KLF2	2.154	3	25.1885±4.6150	270.3634 ± 49.8964	5.297981
DUSP1	2.056	5	26.7865±6.6521	248.5907 ± 44.5847	7.957363
ADAMTS1,4	9.361	9	0.1300±0.0477	12.0537 ± 3.6829	4.648776
FOSB	6.092	5	0.0902±0.049	20.1255 ± 10.4948	5.09732
NR4A1	6.608	6	3.9299±1.8608	34.1467 ± 8.5802	5.057744
CYR61	35.668	8	6.1594±1.3378	45.2667 ± 5.1843	6.545819
JUN	0.941	1	46.8862±5.7799	308.7034 ± 21.8914	9.060202
HES4	1.750	4	16.9848±3.3413	117.2125 ± 23.3421	5.450081
CSRNP1	7.580	4	12.2915±0.9447	92.7416 ± 21.6959	5.641849
DDIT4	1.155	2	13.6124±3.0048	77.4092 ± 11.3541	5.444258
BHLHE40	4.023	5	35.5446±4.4939	170.6737 ± 39.7717	5.672417
CISH	1.276	2	4.0150±0.9245	23.779 ± 6.9188	4.81502
KLF10	6.512	6	7.3659±0.7138	34.7937 ± 8.2337	5.371803
C8orf4	0.317	1	4.3575±0.6835	20.8387 ± 3.823	5.641506
SIK1	11.013	13	5.7743±0.5837	29.7922 ± 7.4416	4.695809
GADD45B	1.303	4	77.2739±16.7875	255.9904 ± 62.5217	3.923338
ETS2	12.372	8	6.3766±0.5978	22.9965 ± 5.6914	3.933248
CDKN1A	1.145	2	14.7643±1.2850	46.8025 ± 10.8005	3.796129
TIPARP, PPBP,PF4	32.270	5	8.1208±0.7957	35.5341 ± 13.5265	3.972794
CXCL	66.294	4	0.4348±0.2901	3.8827 ± 1.5831	3.865725
NFIL3	1.382	1	5.6643±0.8178	17.1098 ± 4.4111	4.197002
C2orf77	1.4315	9	2.1996±0.6032	8.2658 ± 2.9961	4.03729
C10orf10	0.404	2	0.9006±0.2665	4.5674 ± 1.7102	3.870415
SRSF5	6.7568	7	97.0892±3.8894	44.5300 ± 6.3881	4.821116
MAT2A	7.515	9	19.9635±2.1644	8.0140 ± 1.3600	4.254596

Note: Only genes that changed by 2x or more and 0.5x or less were included. Italicized genes decreased expression levels.

General linear model after log_2 transformation and normal/Gaussian distribution with FPR multiple testing correction procedure (ANOVA function in JMP Genomics 5.1).
