# Supplementary material for: Transcriptomic Responses of the Heart and Brain to Anoxia in the Western Painted Turtle
Source: PLoS One. 2015 Jul 6;10(7):e0131669. doi: 10.1371/journal.pone.0131669 (PMC4493013; doi:10.1371/journal.pone.0131669)
Supplement: S4 Table — (PDF) [file pone.0131669.s008.pdf]

S4 Table. Gene Ontology (GO) process-based outputs from genes that were significantly increased in telencephalon of anoxic painted turtles.

Gene Ontology Term	Corrected P-values	FDR Rate	Ortholog
response to organophosphorus	2.31E-07	0.00%	DUSP1, JUNB, EGR1, PTGS2, JUN
response to purine-containing compound	4.98E-07	0.00%	DUSP1, JUNB, EGR1, PTGS2, JUN
response to abiotic stimulus	5.32E-06	0.00%	APOLD1, DUSP1, JUNB, EGR1, PTGS2, DDIT4, JUN
positive regulation of cell death	6.28E-06	0.00%	BTG1, DUSP1, EGR1, PTGS2, DDIT4, JUN
blood vessel development	8.86E-06	0.00%	BTG1, APOLD1, JUNB, EGR1, PTGS2, JUN
vasculature development	1.09E-05	0.00%	BTG1, APOLD1, JUNB, EGR1, PTGS2, JUN
response to cAMP	1.69E-05	0.00%	DUSP1, JUNB, EGR1, JUN
response to radiation	6.70E-05	0.00%	DUSP1, JUNB, EGR1, PTGS2, JUN
cardiovascular system development	0.00012894	0.00%	BTG1, APOLD1, JUNB, EGR1, PTGS2, JUN
circulatory system development	0.00012894	0.00%	BTG1, APOLD1, JUNB, EGR1, PTGS2, JUN
response to mechanical stimulus	0.000179101	0.00%	JUNB, EGR1, PTGS2, JUN
apoptotic process	0.000217119	0.00%	BTG1, DUSP1, EGR1, PTGS2, DDIT4, C8orf4, JUN
blood vessel morphogenesis	0.00022239	0.00%	BTG1, APOLD1, JUNB, PTGS2, JUN
programmed cell death	0.000241201	0.00%	BTG1, DUSP1, EGR1, PTGS2, DDIT4, C8orf4, JUN
tissue development	0.000256321	0.00%	BTG1, APOLD1, DUSP1, JUNB, EGR1, PTGS2, JUN
positive regulation of apoptotic process	0.000270115	0.00%	BTG1, DUSP1, EGR1, PTGS2, JUN
positive regulation of programmed cell death	0.000278519	0.00%	BTG1, DUSP1, EGR1, PTGS2, JUN
cell death	0.00035125	0.00%	BTG1, DUSP1, EGR1, PTGS2, DDIT4, C8orf4, JUN
death	0.00035464	0.00%	BTG1, DUSP1, EGR1, PTGS2, DDIT4, C8orf4, JUN
anatomical structure formation involved in morphogenesis	0.000426741	0.00%	BTG1, APOLD1, DUSP1, JUNB, PTGS2, JUN
positive regulation of neuron death	0.00053526	0.00%	EGR1, DDIT4, JUN
response to hypoxia	0.000667742	0.00%	APOLD1, EGR1, PTGS2, DDIT4
response to decreased oxygen levels	0.000708993	0.00%	APOLD1, EGR1, PTGS2, DDIT4
response to metal ion	0.00096185	0.00%	DUSP1, JUNB, PTGS2, JUN
response to oxygen levels	0.000970699	0.00%	APOLD1, EGR1, PTGS2, DDIT4
response to alcohol	0.001181114	0.00%	DUSP1, JUNB, EGR1, PTGS2
regulation of cell death	0.001750241	0.00%	BTG1, DUSP1, EGR1, PTGS2, DDIT4, JUN
response to organonitrogen compound	0.00175966	0.00%	DUSP1, JUNB, EGR1, PTGS2, JUN

response to lipid	0.001796515	0.00%	DUSP1, JUNB, EGR1, PTGS2, JUN
response to calcium ion	0.002056717	0.00%	DUSP1, JUNB, JUN
response to organic cyclic compound	0.002156366	0.00%	DUSP1, JUNB, EGR1, PTGS2, JUN
response to nitrogen compound	0.002722042	0.00%	DUSP1, JUNB, EGR1, PTGS2, JUN
angiogenesis	0.004480801	0.00%	BTG1, APOLD1, PTGS2, JUN
response to steroid hormone	0.004536262	0.00%	DUSP1, JUNB, EGR1, PTGS2
response to inorganic substance	0.005088813	0.17%	DUSP1, JUNB, PTGS2, JUN
regulation of endothelial cell differentiation	0.005639408	0.17%	BTG1, APOLD1
response to glucocorticoid	0.005649543	0.16%	DUSP1, JUNB, PTGS2
response to drug	0.00568962	0.16%	JUNB, EGR1, PTGS2, JUN
cellular response to endogenous stimulus	0.006573277	0.21%	DUSP1, JUNB, EGR1, PTGS2, JUN
cell proliferation	0.006652234	0.20%	BTG1, JUNB, EGR1, PTGS2, DDIT4, JUN
response to corticosteroid	0.006782327	0.20%	DUSP1, JUNB, PTGS2
positive regulation of developmental process	0.007882197	0.24%	BTG1, JUNB, EGR1, PTGS2, JUN
cellular response to organic substance	0.009369857	0.23%	DUSP1, JUNB, EGR1, PTGS2, DDIT4, JUN

Corrected P-values represent the Simulation Corrected P-values generated from the GO Term Finder.
