# Supplementary material for: Transcriptomic Responses of the Heart and Brain to Anoxia in the Western Painted Turtle
Source: PLoS One. 2015 Jul 6;10(7):e0131669. doi: 10.1371/journal.pone.0131669 (PMC4493013; doi:10.1371/journal.pone.0131669)
Supplement: S5 Table — (PDF) [file pone.0131669.s009.pdf]

S5 Table. Gene Ontology (GO) process-based outputs from genes that were significantly increased in ventricle of anoxic painted turtles.

Gene Ontology Term	Corrected p-values	FDR Rate	Ortholog
response to organophosphorus	1.38E-07	0.00%	DUSP1, EGR1, JUNB, PTGS2, CDO1, JUN
response to purine-containing compound	3.45E-07	0.00%	DUSP1, EGR1, JUNB, PTGS2, CDO1, JUN
circadian rhythm	1.41E-06	0.00%	BHLHE40, EGR1, SIK1, KLF10, NFIL3, JUN
response to radiation	3.34E-06	0.00%	BHLHE40, DUSP1, EGR1, JUNB, PTGS2, SIK1, JUN
response to cAMP	4.32E-06	0.00%	DUSP1, EGR1, JUNB, CDO1, JUN
response to abiotic stimulus	5.21E-06	0.00%	BHLHE40, DDIT4, APOLD1, PTGS2, JUNB, EGR1, DUSP1, SIK1, JUN
response to organonitrogen compound	1.39E-05	0.00%	CISH, PTGS2, JUNB, EGR1, DUSP1, KLF10, JUN, CDO1
rhythmic process	2.12E-05	0.00%	BHLHE40, EGR1, SIK1, KLF10, NFIL3, JUN
response to nitrogen compound	2.77E-05	0.00%	CISH, PTGS2, JUNB, EGR1, DUSP1, KLF10, JUN, CDO1
positive regulation of cell death	3.16E-05	0.00%	BTG1, DUSP1, EGR1, PTGS2, SIK1, DDIT4, JUN
response to oxygen-containing compound	5.92E-05	0.00%	CISH, PPBP, PTGS2, JUNB, EGR1, DUSP1, KLF10, JUN, CDO1
cellular response to organic substance	0.0001042	0.00%	DDIT4, CISH, PPBP, PTGS2, JUNB, EGR1, DUSP1, KLF10, NFIL3, JUN
response to organic substance	0.0001198	0.00%	DDIT4, CISH, PPBP, PTGS2, JUNB, EGR1, DUSP1, KLF10, CDO1, NFIL3, JUN
response to cytokine	0.0001266	0.00%	PPBP, EGR1, JUNB, PTGS2, NFIL3, JUN, CISH
negative regulation of cellular metabolic process	0.000137	0.00%	BHLHE40, DDIT4, CISH, JUNB, EGR1, DUSP1, SIK1, KLF10, NFIL3, JUN
response to lipid	0.0003137	0.00%	PPBP, DUSP1, EGR1, JUNB, PTGS2, CDO1, JUN
negative regulation of metabolic process	0.0004525	0.00%	BHLHE40, DDIT4, CISH, JUNB, EGR1, DUSP1, SIK1, KLF10, NFIL3, JUN
negative regulation of cellular biosynthetic process	0.0004789	0.00%	BHLHE40, JUNB, EGR1, DUSP1, KLF10, SIK1, JUN, NFIL3
negative regulation of biosynthetic process	0.0005251	0.00%	BHLHE40, JUNB, EGR1, DUSP1, KLF10, SIK1, JUN, NFIL3
cellular response to chemical stimulus	0.0005714	0.00%	DDIT4, CISH, PPBP, PTGS2, JUNB, EGR1, DUSP1, KLF10, NFIL3, JUN
positive regulation of apoptotic process	0.0006104	0.00%	BTG1, DUSP1, EGR1, PTGS2, SIK1, JUN
positive regulation of programmed cell death	0.0006328	0.00%	BTG1, DUSP1, EGR1, PTGS2, SIK1, JUN
tissue development	0.0006449	0.00%	BTG1, APOLD1, PTGS2, JUNB, EGR1, DUSP1, KLF10, SIK1, JUN
response to light stimulus	0.0007738	0.00%	BHLHE40, DUSP1, JUNB, PTGS2, SIK1
response to alcohol	0.000844	0.00%	DUSP1, EGR1, JUNB, PTGS2, CDO1
cardiovascular system development	0.000981	0.00%	BTG1, APOLD1, EGR1, JUNB, PTGS2, SIK1, JUN
circulatory system development	0.000981	0.00%	BTG1, APOLD1, EGR1, JUNB, PTGS2, SIK1, JUN

negative regulation of cellular process	0.0010839	0.00%	BHLHE40, BTG1, DDIT4, CISH, PTGS2, JUNB, EGR1, DUSP1, SIK1, KLF10, NFIL3, JUN
blood vessel development	0.0011147	0.00%	BTG1, APOLD1, EGR1, JUNB, PTGS2, JUN
response to glucocorticoid	0.0011155	0.00%	DUSP1, JUNB, PTGS2, CDO1
vasculature development	0.0013579	0.00%	BTG1, APOLD1, EGR1, JUNB, PTGS2, JUN
negative regulation of macromolecule metabolic process	0.0013756	0.00%	BHLHE40, DDIT4, CISH, JUNB, EGR1, DUSP1, KLF10, NFIL3, JUN
response to corticosteroid	0.0014219	0.00%	DUSP1, JUNB, PTGS2, CDO1
response to external stimulus	0.0014425	0.00%	BHLHE40, DDIT4, PPBP, PTGS2, JUNB, EGR1, KLF10, SIK1, JUN
response to endogenous stimulus	0.0016575	0.00%	CISH, PTGS2, JUNB, EGR1, DUSP1, KLF10, JUN, CDO1
cellular response to endogenous stimulus	0.0018508	0.00%	DUSP1, EGR1, JUNB, PTGS2, KLF10, JUN, CISH
enzyme linked receptor protein signaling pathway	0.0019813	0.00%	CSRNP1, EGR1, JUNB, KLF10, DDIT4, JUN, CISH
negative regulation of biological process	0.0027186	0.00%	BHLHE40, BTG1, DDIT4, CISH, PTGS2, JUNB, EGR1, DUSP1, SIK1, KLF10, NFIL3, JUN
response to mechanical stimulus	0.0039143	0.00%	EGR1, JUNB, PTGS2, JUN
negative regulation of nucleobase-containing compound metabolic process	0.0043458	0.00%	BHLHE40, DUSP1, EGR1, JUNB, KLF10, NFIL3, JUN
response to steroid hormone	0.0044293	0.00%	DUSP1, EGR1, JUNB, PTGS2, CDO1
response to chemical	0.0048851	0.00%	DDIT4, CISH, PPBP, PTGS2, JUNB, EGR1, DUSP1, KLF10, CDO1, NFIL3, JUN
negative regulation of macromolecule biosynthetic process	0.0050162	0.00%	BHLHE40, DUSP1, EGR1, JUNB, KLF10, NFIL3, JUN
negative regulation of nitrogen compound metabolic process	0.0050344	0.00%	BHLHE40, DUSP1, EGR1, JUNB, KLF10, NFIL3, JUN
positive regulation of neuron death	0.0053788	0.00%	EGR1, DDIT4, JUN
single-multicellular organism process	0.0056246	0.00%	BHLHE40, BTG1, DDIT4, PPBP, APOLD1, CSRNP1, PTGS2, JUNB, EGR1, DUSP1, SIK1, KLF10, CDO1, JUN
apoptotic process	0.0060689	0.00%	BTG1, DDIT4, CSRNP1, PTGS2, EGR1, DUSP1, SIK1, JUN
response to peptide	0.0067741	0.00%	EGR1, JUNB, KLF10, CDO1, CISH
programmed cell death	0.006796	0.00%	BTG1, DDIT4, CSRNP1, PTGS2, EGR1, DUSP1, SIK1, JUN
response to organic cyclic compound	0.0069322	0.00%	DUSP1, EGR1, JUNB, PTGS2, CDO1, JUN
cell proliferation	0.0086096	0.00%	BTG1, DDIT4, PPBP, PTGS2, JUNB, EGR1, KLF10, JUN
multicellular organismal process	0.0090392	0.00%	BHLHE40, BTG1, DDIT4, PPBP, APOLD1, CSRNP1, PTGS2, JUNB, EGR1, DUSP1, SIK1, KLF10, CDO1, JUN

Corrected P-values represent the Simulation Corrected P-values generated from the GO Term Finder.
