# Supplementary material for: Transcriptomic Responses of the Heart and Brain to Anoxia in the Western Painted Turtle
Source: PLoS One. 2015 Jul 6;10(7):e0131669. doi: 10.1371/journal.pone.0131669 (PMC4493013; doi:10.1371/journal.pone.0131669)
Supplement: S6 Table — (PDF) [file pone.0131669.s010.pdf]

S6 Table. Gene Ontology (GO) process-based outputs from genes that were significantly increased across tissues (treatment only effects) in anoxic painted turtles.

Gene Ontology Term	Corrected p-values	FDR Rate	Ortholog
response to radiation	8.63E-08	0.00%	BHLHE40, PTGS2, EGR1, JUNB, DUSP1, SIK1, CDKN1A, JUN
response to abiotic stimulus	2.39E-07	0.00%	BHLHE40, DDIT4, APOLD1, PTGS2, EGR1, JUNB, DUSP1, CDKN1A, SIK1, JUN
positive regulation of cell death	1.14E-06	0.00%	BTG1, DDIT4, PTGS2, EGR1, DUSP1, SIK1, CDKN1A, JUN
circadian rhythm	1.58E-06	0.00%	BHLHE40, EGR1, SIK1, KLF10, NFIL3, JUN
cellular response to chemical stimulus	3.59E-06	0.00%	DDIT4, CISH, PPBP, PTGS2, EGR1, JUNB, DUSP1, CDKN1A, KLF10, MAT2A, NFIL3, JUN
cellular response to organic substance	7.87E-06	0.00%	DDIT4, CISH, PPBP, PTGS2, EGR1, JUNB, DUSP1, CDKN1A, KLF10, NFIL3, JUN
negative regulation of cellular metabolic process	1.07E-05	0.00%	BHLHE40, DDIT4, CISH, EGR1, JUNB, DUSP1, CDKN1A, SIK1, KLF10, NFIL3, JUN
response to organophosphorus	1.39E-05	0.00%	DUSP1, JUNB, EGR1, PTGS2, JUN
response to organonitrogen compound	1.55E-05	0.00%	CISH, PTGS2, EGR1, JUNB, DUSP1, KLF10, CDKN1A, JUN
response to light stimulus	2.25E-05	0.00%	BHLHE40, DUSP1, JUNB, PTGS2, CDKN1A, SIK1
rhythmic process	2.37E-05	0.00%	BHLHE40, EGR1, SIK1, KLF10, NFIL3, JUN
positive regulation of programmed cell death	2.69E-05	0.00%	BTG1, DUSP1, EGR1, PTGS2, CDKN1A, SIK1, JUN
response to purine-containing compound	2.98E-05	0.00%	DUSP1, JUNB, EGR1, PTGS2, JUN
response to nitrogen compound	3.10E-05	0.00%	CISH, PTGS2, EGR1, JUNB, DUSP1, KLF10, CDKN1A, JUN
negative regulation of metabolic process	4.04E-05	0.00%	BHLHE40, DDIT4, CISH, EGR1, JUNB, DUSP1, CDKN1A, SIK1, KLF10, NFIL3, JUN
apoptotic process	4.24E-05	0.00%	BTG1, DDIT4, C8orf4, CSRNP1, PTGS2, EGR1, DUSP1, CDKN1A, SIK1, JUN
programmed cell death	4.91E-05	0.00%	BTG1, DDIT4, C8orf4, CSRNP1, PTGS2, EGR1, DUSP1, CDKN1A, SIK1, JUN
tissue development	5.34E-05	0.00%	BTG1, APOLD1, PTGS2, EGR1, JUNB, DUSP1, CDKN1A, KLF10, SIK1, JUN
response to oxygen-containing compound	6.62E-05	0.00%	CISH, PPBP, PTGS2, EGR1, JUNB, DUSP1, KLF10, CDKN1A, JUN
cell death	8.25E-05	0.00%	BTG1, DDIT4, C8orf4, CSRNP1, PTGS2, EGR1, DUSP1, CDKN1A, SIK1, JUN
death	8.36E-05	0.00%	BTG1, DDIT4, C8orf4, CSRNP1, PTGS2, EGR1, DUSP1, CDKN1A, SIK1, JUN
negative regulation of cellular process	0.000123039	0.00%	BHLHE40, BTG1, DDIT4, CISH, PTGS2, EGR1, JUNB, DUSP1, CDKN1A, SIK1, KLF10, NFIL3, JUN
cellular response to endogenous stimulus	0.000124553	0.00%	CISH, PTGS2, EGR1, JUNB, DUSP1, KLF10, CDKN1A, JUN
negative regulation of macromolecule metabolic process	0.000125356	0.00%	BHLHE40, DDIT4, CISH, EGR1, JUNB, DUSP1, CDKN1A, KLF10, NFIL3, JUN
response to external stimulus	0.00013226	0.00%	BHLHE40, DDIT4, PPBP, PTGS2, EGR1, JUNB, CDKN1A, KLF10, SIK1, JUN
response to organic substance	0.000133935	0.00%	DDIT4, CISH, PPBP, PTGS2, EGR1, JUNB, DUSP1, CDKN1A, KLF10, NFIL3, JUN
enzyme linked receptor protein signaling pathway	0.000134754	0.00%	DDIT4, CISH, CSRNP1, EGR1, JUNB, KLF10, CDKN1A, JUN

response to cytokine	0.000141501	0.00%	PPBP, JUNB, EGR1, PTGS2, NFIL3, CISH, JUN
negative regulation of biological process	0.000340106	0.00%	BHLHE40, BTG1, DDIT4, CISH, PTGS2, EGR1, JUNB, DUSP1, CDKN1A, SIK1, KLF10, NFIL3, JUN
response to lipid	0.000350716	0.00%	PPBP, DUSP1, JUNB, EGR1, PTGS2, CDKN1A, JUN
response to cAMP	0.000423106	0.00%	DUSP1, JUNB, EGR1, JUN
cellular response to external stimulus	0.000471987	0.00%	EGR1, PTGS2, CDKN1A, KLF10, JUN
negative regulation of cellular biosynthetic process	0.000535471	0.00%	BHLHE40, EGR1, JUNB, DUSP1, KLF10, SIK1, JUN, NFIL3
negative regulation of biosynthetic process	0.000587145	0.00%	BHLHE40, EGR1, JUNB, DUSP1, KLF10, SIK1, JUN, NFIL3
response to chemical	0.000620845	0.00%	DDIT4, CISH, PPBP, PTGS2, EGR1, JUNB, DUSP1, CDKN1A, KLF10, MAT2A, NFIL3, JUN
negative regulation of protein phosphorylation	0.000637771	0.00%	DUSP1, CDKN1A, DDIT4, CISH, JUN
positive regulation of apoptotic process	0.000682503	0.00%	BTG1, DUSP1, EGR1, PTGS2, SIK1, JUN
response to oxygen levels	0.00074048	0.00%	APOLD1, EGR1, PTGS2, CDKN1A, DDIT4
cell proliferation	0.000876997	0.00%	BTG1, DDIT4, PPBP, PTGS2, EGR1, JUNB, KLF10, CDKN1A, JUN
response to alcohol	0.000943657	0.00%	DUSP1, JUNB, EGR1, PTGS2, CDKN1A
cardiovascular system development	0.00109685	0.00%	BTG1, APOLD1, JUNB, EGR1, PTGS2, SIK1, JUN
circulatory system development	0.00109685	0.00%	BTG1, APOLD1, JUNB, EGR1, PTGS2, SIK1, JUN
blood vessel development	0.001246348	0.00%	BTG1, APOLD1, JUNB, EGR1, PTGS2, JUN
response to glucocorticoid	0.001247299	0.00%	DUSP1, JUNB, PTGS2, CDKN1A
vasculature development	0.001518325	0.00%	BTG1, APOLD1, JUNB, EGR1, PTGS2, JUN
cellular response to growth factor stimulus	0.001566417	0.00%	JUNB, EGR1, CDKN1A, KLF10, DDIT4, JUN
response to corticosteroid	0.001589837	0.00%	DUSP1, JUNB, PTGS2, CDKN1A
regulation of cell death	0.001749564	0.04%	BTG1, DDIT4, PTGS2, EGR1, DUSP1, SIK1, CDKN1A, JUN
response to endogenous stimulus	0.00185326	0.08%	CISH, PTGS2, EGR1, JUNB, DUSP1, KLF10, CDKN1A, JUN
response to growth factor	0.001893184	0.08%	JUNB, EGR1, CDKN1A, KLF10, DDIT4, JUN
positive regulation of cellular process	0.001957781	0.08%	BTG1, DDIT4, PPBP, CSRNP1, PTGS2, EGR1, JUNB, DUSP1, CDKN1A, SIK1, KLF10, JUN
negative regulation of phosphorylation	0.001967617	0.08%	DUSP1, CDKN1A, DDIT4, CISH, JUN
positive regulation of biological process	0.002079451	0.08%	BTG1, DDIT4, PPBP, CSRNP1, PTGS2, EGR1, JUNB, DUSP1, CDKN1A, SIK1, KLF10, NFIL3, JUN
regulation of primary metabolic process	0.002821464	0.07%	BHLHE40, BTG1, DDIT4, CISH, CSRNP1, PTGS2, EGR1, JUNB, DUSP1, CDKN1A, SIK1, KLF10, NFIL3, JUN
cellular nitrogen compound biosynthetic process	0.002874041	0.07%	BHLHE40, BTG1, CSRNP1, PTGS2, EGR1, JUNB, DUSP1, CDKN1A, KLF10, SIK1, MAT2A, NFIL3, JUN
negative regulation of protein modification process	0.003625206	0.07%	DUSP1, CDKN1A, DDIT4, CISH, JUN
regulation of cellular metabolic process	0.003865864	0.07%	BHLHE40, BTG1, DDIT4, CISH, CSRNP1, PTGS2, EGR1, JUNB, DUSP1, CDKN1A, SIK1, KLF10, NFIL3, JUN
response to mechanical stimulus	0.004376652	0.07%	JUNB, EGR1, PTGS2, JUN

negative regulation of nucleobase-containing compound metabolic process	0.004859207	0.07%	BHLHE40, DUSP1, JUNB, EGR1, KLF10, NFIL3, JUN
response to steroid hormone	0.004952476	0.07%	DUSP1, JUNB, EGR1, PTGS2, CDKN1A
negative regulation of phosphorus metabolic process	0.005065543	0.10%	DUSP1, CDKN1A, DDIT4, CISH, JUN
negative regulation of phosphate metabolic process	0.005065543	0.10%	DUSP1, CDKN1A, DDIT4, CISH, JUN
negative regulation of macromolecule biosynthetic process	0.005608712	0.10%	BHLHE40, DUSP1, JUNB, EGR1, KLF10, NFIL3, JUN
negative regulation of nitrogen compound metabolic process	0.00562913	0.09%	BHLHE40, DUSP1, JUNB, EGR1, KLF10, NFIL3, JUN
response to extracellular stimulus	0.005829462	0.09%	EGR1, PTGS2, CDKN1A, KLF10, JUN
positive regulation of neuron death	0.006014223	0.09%	EGR1, DDIT4, JUN
single-multicellular organism process	0.006289066	0.12%	BHLHE40, BTG1, DDIT4, PPBP, APOLD1, CSRNP1, PTGS2, EGR1, JUNB, DUSP1, CDKN1A, SIK1, KLF10, JUN
response to drug	0.006540306	0.12%	JUNB, EGR1, PTGS2, CDKN1A, JUN
response to stimulus	0.007492097	0.14%	BHLHE40, PPBP, APOLD1, CSRNP1, JUNB, CDKN1A, SIK1, KLF10, NFIL3, JUN, DDIT4, CISH, DUSP1, EGR1, PTGS2, MAT2A
response to organic cyclic compound	0.007751101	0.14%	DUSP1, JUNB, EGR1, PTGS2, CDKN1A, JUN
regulation of nitrogen compound metabolic process	0.008367551	0.14%	BHLHE40, BTG1, CSRNP1, PTGS2, EGR1, JUNB, DUSP1, CDKN1A, KLF10, SIK1, NFIL3, JUN
regulation of cellular biosynthetic process	0.009271878	0.14%	BHLHE40, BTG1, CSRNP1, PTGS2, EGR1, JUNB, DUSP1, CDKN1A, KLF10, SIK1, NFIL3, JUN

Corrected P-values represent the Simulation Corrected P-values generated from the GO Term Finder.
