# Supplementary material for: Transcriptomic Responses of the Heart and Brain to Anoxia in the Western Painted Turtle
Source: PLoS One. 2015 Jul 6;10(7):e0131669. doi: 10.1371/journal.pone.0131669 (PMC4493013; doi:10.1371/journal.pone.0131669)
Supplement: S7 Table — (PDF) [file pone.0131669.s011.pdf]

S7 Table. Gene Ontology (GO) function-based outputs from genes that were significantly increased in telencephalon of anoxic painted turtles.

Gene Ontology Term	Corrected p-values	FDR Rate	Orthologs
double-stranded DNA binding	0.0005089	0.00%	JUNB, EGR1, JUN
structure-specific DNA binding	0.0040734	0.00%	JUNB, EGR1, JUN
RNA polymerase II regulatory region sequence-specific DNA binding	0.0077373	0.00%	JUNB, EGR1, JUN
RNA polymerase II regulatory region DNA binding	0.0080879	0.00%	JUNB, EGR1, JUN

Corrected P-values represent the Simulation Corrected P-values generated from the GO Term Finder.
