# Supplementary material for: Transcriptomic Responses of the Heart and Brain to Anoxia in the Western Painted Turtle
Source: PLoS One. 2015 Jul 6;10(7):e0131669. doi: 10.1371/journal.pone.0131669 (PMC4493013; doi:10.1371/journal.pone.0131669)
Supplement: S8 Table — (PDF) [file pone.0131669.s012.pdf]

S8 Table. Gene Ontology (GO) function-based outputs from genes that were significantly increased in ventricle of anoxic painted turtles.

Gene Ontology Term	Corrected p-values	FDR Rate	Ortholog
transcription regulatory region DNA binding	0.0001339	0.00%	BHLHE40, EGR1, JUNB, KLF10, NFIL3, JUN
regulatory region DNA binding	0.0001715	0.00%	BHLHE40, EGR1, JUNB, KLF10, NFIL3, JUN
regulatory region nucleic acid binding	0.0001715	0.00%	BHLHE40, EGR1, JUNB, KLF10, NFIL3, JUN
transcription regulatory region sequence-specific DNA binding	0.0005125	0.00%	EGR1, JUNB, KLF10, NFIL3, JUN
sequence-specific DNA binding RNA polymerase II transcription factor activity	0.0010772	0.00%	BHLHE40, CSRNP1, EGR1, NFIL3, JUN
transcription cofactor activity	0.0013604	0.00%	BHLHE40, BTG1, JUNB, NFIL3, JUN
RNA polymerase II core promoter proximal region sequence-specific DNA binding transcription factor activity	0.0013744	0.00%	BHLHE40, EGR1, NFIL3, JUN
nucleic acid binding transcription factor activity	0.0015526	0.00%	BHLHE40, CSRNP1, EGR1, JUNB, KLF10, NFIL3, JUN
sequence-specific DNA binding transcription factor activity	0.0015526	0.00%	BHLHE40, CSRNP1, EGR1, JUNB, KLF10, NFIL3, JUN
sequence-specific DNA binding	0.0018632	0.00%	CSRNP1, EGR1, JUNB, KLF10, NFIL3, JUN
transcription factor binding transcription factor activity	0.0020989	0.18%	BHLHE40, BTG1, JUNB, NFIL3, JUN
protein binding transcription factor activity	0.0021343	0.17%	BHLHE40, BTG1, JUNB, NFIL3, JUN
core promoter sequence-specific DNA binding	0.0024125	0.15%	EGR1, KLF10, NFIL3
RNA polymerase II regulatory region sequence-specific DNA binding	0.005272	0.43%	EGR1, JUNB, NFIL3, JUN
RNA polymerase II regulatory region DNA binding	0.0055877	0.40%	EGR1, JUNB, NFIL3, JUN
14-3-3 protein binding	0.0058523	0.50%	SIK1, DDIT4
core promoter binding	0.0068916	0.47%	EGR1, KLF10, NFIL3
double-stranded DNA binding	0.0070891	0.44%	EGR1, JUNB, JUN
RNA polymerase II activating transcription factor binding	0.0078472	0.42%	BHLHE40, JUN

Corrected P-values represent the Simulation Corrected P-values generated from the GO Term Finder.
