# Supplementary material for: Transcriptomic Responses of the Heart and Brain to Anoxia in the Western Painted Turtle
Source: PLoS One. 2015 Jul 6;10(7):e0131669. doi: 10.1371/journal.pone.0131669 (PMC4493013; doi:10.1371/journal.pone.0131669)
Supplement: S3 Fig — Smaller, darker nodes correlate to the lowest P-values measured for the set of GO terms. Branches between nodes reflect predicted network associations, with the thickness of branches reflecting strength of association. (PDF) [file pone.0131669.s003.pdf]

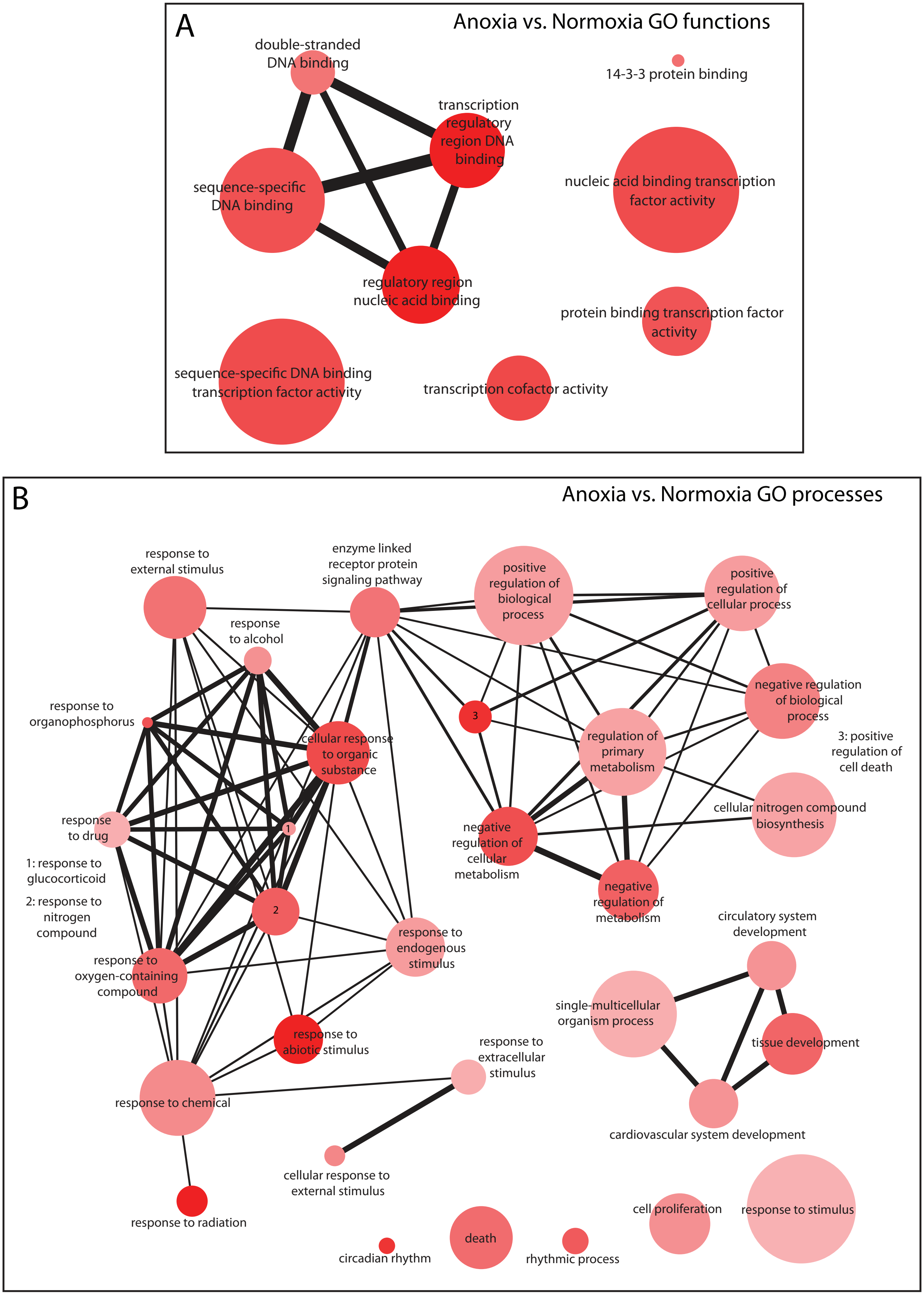
A
Anoxia vs. Normoxia GO functions
double-stranded DNA binding
14-3-3 protein binding
transcription regulatory region DNA binding
sequence-specific DNA binding
nucleic acid binding transcription factor activity
regulatory region nucleic acid binding
protein binding transcription factor activity
sequence-specific DNA binding transcription factor activity
transcription cofactor activity
B
Anoxia vs. Normoxia GO processes
response to external stimulus
enzyme linked receptor protein signaling pathway
positive regulation of biological process
positive regulation of cellular process
response to alcohol
response to organophosphorus
negative regulation of biological process
3
cellular response to organic substance
regulation of primary metabolism
3: positive regulation of cell death
response to drug
1
cellular nitrogen compound biosynthesis
negative regulation of cellular metabolism
1: response to glucocorticoid
2: response to nitrogen compound
2
negative regulation of metabolism
circulatory system development
response to endogenous stimulus
response to oxygen-containing compound
single-multicellular organism process
response to abiotic stimulus
tissue development
response to extracellular stimulus
response to chemical
cardiovascular system development
cellular response to external stimulus
response to radiation
cell proliferation
response to stimulus
death
circadian rhythm
rhythmic process
