# Supplementary material for: Transcriptomic Responses of the Heart and Brain to Anoxia in the Western Painted Turtle
Source: PLoS One. 2015 Jul 6;10(7):e0131669. doi: 10.1371/journal.pone.0131669 (PMC4493013; doi:10.1371/journal.pone.0131669)
Supplement: S9 Table — (PDF) [file pone.0131669.s013.pdf]

S9 Table. Gene Ontology (GO) function-based outputs from genes that were significantly increased across tissues (treatment only effects) in anoxic painted turtles.

Gene Ontology Term	Corrected P-values	FDR Rate	Ortholog
transcription regulatory region DNA binding	0.0001793	0.00%	BHLHE40, JUNB, EGR1, KLF10, NFIL3, JUN
regulatory region DNA binding	0.0002296	0.00%	BHLHE40, JUNB, EGR1, KLF10, NFIL3, JUN
regulatory region nucleic acid binding	0.0002296	0.00%	BHLHE40, JUNB, EGR1, KLF10, NFIL3, JUN
transcription regulatory region sequence-specific DNA binding	0.0006862	0.00%	JUNB, EGR1, KLF10, NFIL3, JUN
sequence-specific DNA binding RNA polymerase II transcription factor activity	0.0014424	0.00%	BHLHE40, CSRNP1, EGR1, NFIL3, JUN
transcription cofactor activity	0.0018216	0.00%	BHLHE40, BTG1, JUNB, NFIL3, JUN
RNA polymerase II core promoter proximal region sequence-specific DNA binding transcription factor activity	0.0018403	0.00%	BHLHE40, EGR1, NFIL3, JUN
nucleic acid binding transcription factor activity	0.0020788	0.00%	BHLHE40, CSRNP1, JUNB, EGR1, KLF10, NFIL3, JUN
sequence-specific DNA binding transcription factor activity	0.0020788	0.00%	BHLHE40, CSRNP1, JUNB, EGR1, KLF10, NFIL3, JUN
sequence-specific DNA binding	0.0024948	0.00%	CSRNP1, JUNB, EGR1, KLF10, NFIL3, JUN
transcription factor binding transcription factor activity	0.0028104	0.00%	BHLHE40, BTG1, JUNB, NFIL3, JUN
protein binding transcription factor activity	0.0028578	0.00%	BHLHE40, BTG1, JUNB, NFIL3, JUN
core promoter sequence-specific DNA binding	0.0032303	0.00%	EGR1, KLF10, NFIL3
RNA polymerase II regulatory region sequence-specific DNA binding	0.0070591	0.00%	JUNB, EGR1, NFIL3, JUN
RNA polymerase II regulatory region DNA binding	0.0074818	0.00%	JUNB, EGR1, NFIL3, JUN
14-3-3 protein binding	0.0078361	0.00%	SIK1, DDIT4
core promoter binding	0.0092277	0.00%	EGR1, KLF10, NFIL3
double-stranded DNA binding	0.0094921	0.00%	JUNB, EGR1, JUN

Corrected P-values represent the Simulation Corrected P-values generated from the GO Term Finder.
